# Targeting adipocyte ESRRA alleviates osteoarthritis via interrupting inter-organelle crosstalk of complement C3-CFD-MAC cascade

**DOI:** 10.1038/s41413-026-00527-3

**Published:** 2026-04-29

**Authors:** Tongling Huang, Zihui Wang, Lu Gao, Jun Gao, Zhaocheng Lu, Pengda Li, Chon Him Choy, Zhuolei Yuan, Yanting Zhong, Chang-An Geng, Huaiyu Wang, Kelvin W. K. Yeung, Bin Li, Haobo Pan, Di Chen, Min Guan

**Affiliations:** 1 Shenzhen Key Laboratory of Marine Biomaterials, Center for Human Tissues and Organs Degeneration, Institute of Biomedicine and Biotechnology, Shenzhen Institutes of Advanced Technology, Chinese Academy of Sciences, Shenzhen, China; 2 University of Chinese Academy of Sciences, Beijing, China; 3 Division of Biosciences, University College London, London, UK; 4 State Key Laboratory of Phytochemistry and Plant Resources in West China, Kunming Institute of Botany, Chinese Academy of Sciences, Kunming, China; 5 Center for AI-Driven Medical Research, Institute of Biomedicine and Biotechnology, Shenzhen Institutes of Advanced Technology, Chinese Academy of Sciences, Shenzhen, China; 6 Department of Orthopaedics and Traumatology, Li Ka Shing Faculty of Medicine, The University of Hong Kong, Hong Kong, China; 7 Department of Orthopaedics and Traumatology, The University of Hong Kong-Shenzhen Hospital, Shenzhen, China; 8 Medical 3D Printing Center, Orthopedic Institute, Department of Orthopedic Surgery, The First Affiliated Hospital, School of Basic Medical Sciences, Interdisciplinary Innovation Center for Nanomedicine, MOE Key Laboratory of Geriatric Diseases and Immunology, Suzhou Medical College, Soochow University, Suzhou, China; 9 Faculty of Pharmaceutical Sciences, Shenzhen University of Advanced Technology, Shenzhen, Guangdong China

**Keywords:** Bone, Endocrine system and metabolic diseases, Fat metabolism, Metabolic syndrome, Metabolic disorders

## Abstract

Osteoarthritis is an aging-related systemic disease involving the crosstalk of multiple organs/tissues in metabolism and inflammation, yet little is known about the contribution of liver and marrow adipose tissue (MAT). Here we show that MAT-derived complement factor D (CFD) and component 3 (C3) derived from steatotic liver coordinately drive excessive alternative complement activation, resulting in cartilage damage in mice during aging and metabolic disorders. Mechanistically, estrogen-related receptor α (ESRRA) transcriptionally upregulates CFD responding to bone marrow adipocytes (BMAds) expansion. Inhibition of ESRRA/CFD signaling in BMAds blocks the chondrocyte senescence and catabolism triggered by C3 that is released from steatotic hepatocyte, interrupting C3-CFD-MAC cascade, thereby suppressing ERK1/2 phosphorylation and mitochondrial dysfunction. Adipocyte-specific ablation or pharmacological inhibition of ESRRA reduces CFD levels particularly in adipocyte-rich bone marrow, attenuating osteoarthritis progression in aged mice. Our findings highlight a key liver-MAT-cartilage axis bridged by C3-CFD-MAC pathway, raising the potential for adipocyte ESRRA-targeting therapies for aging-related metabolic osteoarthritis.

## Introduction

Skeletal aging is closely intertwined with metabolic disorders and low-grade chronic inflammation, driving pathogenesis such as osteoarthritis and osteoporosis that frequently exist as comorbidities.^1^ Recognized as a clinically and etiologically heterogeneous disorder, osteoarthritis affects nearly 500 million individuals worldwide, yet lacks disease-modifying pharmacotherapies.^2^ Most therapeutic development for osteoarthritis are currently focused on intra-articular injections, but none can effectively prevent the disease development. Beyond a local tear and wear disease, osteoarthritis progression is reshaped by the central concepts of ‘metaflammation’ and ‘inflammaging’, reflecting an intricate and dynamic interplay of metabolic and immunological factors across multiple organs or tissues.^3,4^ For example, a recent study establishes a functional gut-joint axis through which gut microbial metabolites influence osteoarthritis progression.^5^ Nevertheless, the underlying mechanisms of inter-organ communication in the onset and progression of osteoarthritis during aging or metabolic disorders remain poorly understood.

Aging and metabolic disorder are the key risk factors for osteoarthritis. An escalating incidence of osteoarthritis occurring in middle-aged people is correlated with a global surge in metabolic conditions such as obesity, diabetes, and dyslipidemia, indicating that metabolic syndrome exacerbates the occurrence and progression of osteoarthritis.^1,2,6,7^ Liver is a well-known central organ in the systemic regulation of lipid and carbohydrate metabolism. Clinically, dyslipidemia, hepatic steatosis and adipose tissue expansion often occur as common pathological features in metabolic disorders coupled with high risk factors, including obesity, aging and menopause.^8–11^ Among these metabolic features, systemic excess cholesterol has been well established to contribute to the pathogenesis of metabolic dysfunction-associated steatotic liver disease/steatohepatitis (MASLD/MASH); and also recognized as an independent risk factor for osteoarthritis, rendering chondrocytes sensitive to premature senescence and apoptosis.^7,12,13^ Furthermore, the incidence of MASLD increases with age, leading to hepatocellular senescence that consequently spreads systemic multi-organ senescence.^14,15^ Moreover, hepatokines or extracellular vesicles secreted from injured liver have been characterized to modulate bone homeostasis through the liver-bone axis under diverse conditions, including MASLD/MASH, liver cirrhosis and aging.^16^ These observations suggest that liver dysfunction plays detrimental impacts on multiple organs, including distal skeletal system. Notably, recent studies have identified a positive correlation between MASLD and the development of osteoarthritis in patients.^17–19^ However, it remains unclear whether the liver dysfunction exacerbates osteoarthritis progression via forming a vicious cycle with extrahepatic tissues under systemic stress.

By dynamically coordinating with the liver, adipose tissues function as a crucial metabolic hub regulating systemic energy homeostasis. Importantly, adipose tissue as a linchpin of organismal aging is also a critical regulator of osteoarthritis.^20,21^ Emerging evidence shows that elevated serum and synovial fluid levels of adipokines such as leptin derived from either visceral white adipose tissue (WAT) or joint WAT (also known as infrapatellar fat pad) contribute to osteoarthritis progression in patients and rodents.^22–25^ On the other hand, marrow adipose tissue (MAT) has been recently considered as a true endocrine and paracrine organ with functions far beyond passive storage of energy.^9,26^ In humans and rodent models, MAT expands in response to several established risk factors for skeletal disorders, including aging, obesity, menopause, diabetes, glucocorticoid treatment, and caloric restriction.^9^ For example, pathological accumulation of bone marrow adipocytes (BMAds) serves as a key reservoir of circulating factors including leptin, nuclear factor kappa-B ligand and complement factor D (CFD, also known as adipsin) in rodents and humans, contributing to bone deterioration.^26–29^ However, MAT as a unique fat depot has been largely overlooked in the context of aging-related or metabolic-associated osteoarthritis. Two recent studies describe that BMAds accumulation is positively correlated with cartilage degradation in mice that underwent meniscectomy surgery or fed a high fat diet, implying that BMAds might be new cellular partners involved in osteoarthritis progression.^30,31^ Furthermore, human primary BMAds exhibit a cholesterol-oriented metabolism distinct from peripheral WAT.^32^ Importantly, the transcriptomic profile of human primary adipocytes isolated from epiphyseal bone marrow of osteoarthritic patients suggest a potential role for these BMAds in osteoarthritis development.^31^ However, the reciprocal relationship between joint, MAT and liver remains largely unexplored, with the molecular mechanism governing their cross-talk yet to be elucidated.

We previously characterized that estrogen-related receptor α (ESRRA; also known as ERRα or NR3B1) is a critical modulator of MAT expansion.^29^ As a known druggable nuclear receptor, ESRRA governs the expression of target genes involved in numerous metabolic pathways, which are required in stress-induced response to fasting, calorie restriction or overnutrition.^29,33^ Additionally, cholesterol is considered an ESRRA ligand that activates its transcriptional activity involved in liver function and bone homeostasis.^34–36^ Here, we investigated the effects of ESRRA in adipocytes on joint integrity using two distinct models: a mouse model of chronological aging and an osteoarthritis model induced by overfeeding with a high-fat and high-cholesterol diet (HFHC) combined with surgical destabilization of the medial meniscus (DMM).^12^ Both models develop osteoarthritis-like pathology, accompanied with steatotic liver and high marrow adiposity as well as osteoporotic bone. We show that the complement components C3 and CFD, released predominantly from the steatotic liver and expanded MAT, respectively, coordinately drive chondrocyte senescence and cartilage degeneration via a C3-CFD-MAC (membrane attack complex) cascade. Targeting adipocyte ESRRA counteracts MAT expansion but not peripheral fat pads, and alleviates both spontaneous and experimental osteoarthritis through suppressing the activation of such alternative complement pathway.

## Results

### Conditional adipocyte ablation of *Esrra* counteracts MAT expansion and mitigates spontaneous osteoarthritis in aged mice

**Fig. 1:**
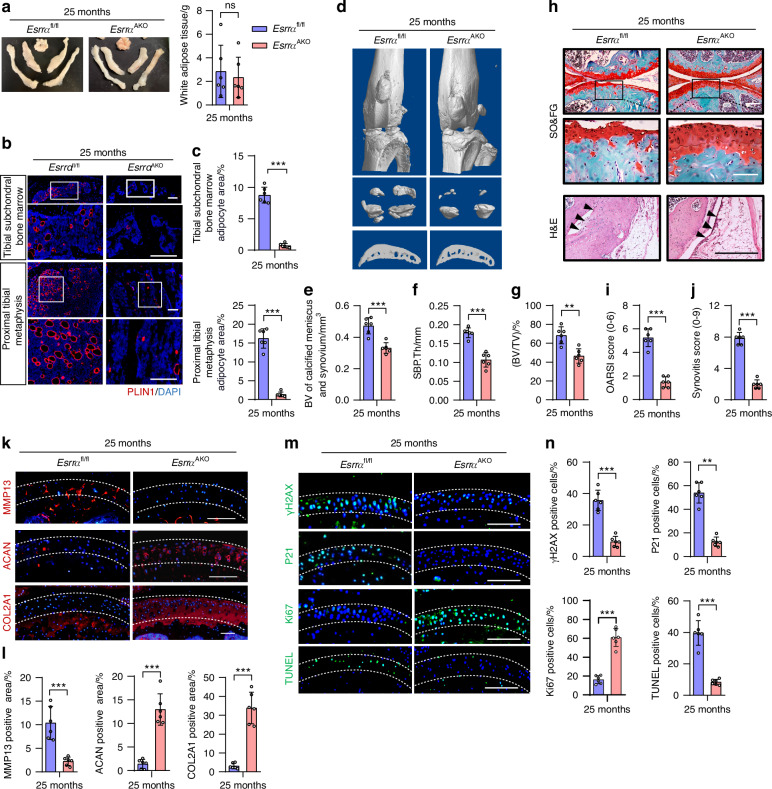
Genetic ablation of *Esrra* in adipocytes counteracts MAT expansion and the pathogenesis of spontaneous osteoarthritis in aged mice. **a** Representative images and weight analysis of peritoneal white adipose tissue (WAT) depots from 25-month-old male *Esrra*^fl/fl^ and *Esrra*^AKO^ mice. **b** Immunofluorescence analysis of Perilipin1-positive (PLIN1^+^) bone marrow adipocytes within the tibial subchondral bone marrow cavity and proximal tibial metaphysis of 25-month-old *Esrra*^fl/fl^ and *Esrra*^AKO^ mice. Scale bar, 100 μm. **c** The area of PLIN1^+^ bone marrow adipocytes was quantified based on the sections in (**b**). **d** Three-dimensional reconstructions of knee joints from microCT scans. **e**–**g** Quantitative analysis of the volume of the calcified meniscus and synovium (**e**), the thickness of subchondral bone plate (SBP) (**f**), and the bone volume/tissue volume ratio (BV/TV) (**g**). **h** Representative images of Safranin O/Fast Green (SO/FG)-stained articular cartilage and H&E-stained synovium. The black arrows point to the synovium. Scale bar, 100 μm. **i**, **j** The Osteoarthritis Research Society International (OARSI) score (**i**) and synovitis score (**j**) were evaluated using established histopathological scoring systems. **k** Immunofluorescence staining for MMP13, ACAN and COL2A1 in articular cartilage. Scale bar, 100 μm. **l** Percentage of MMP13, ACAN, and COL2A1 positive areas within the cartilage area (as indicated by the white dotted line). **m**, **n** Immunofluorescence staining for γH2AX, p21, Ki67 and TUNEL in articular cartilage (**m**) and quantification of positive cells relative to total DAPI-stained cells (**n**). Scale bar, 100 μm. For all experiments, *n* = 6 mice per group. Data are shown as mean ± SD. Statistical analysis is performed using two-sided unpaired Student’s *t*-test

By utilization of mice bearing conditional alleles of *Esrra* and Adiponectin^Cre^ (*Esrra*^fl/fl^; *Adipoq*^Cre^, denoted *Esrra*^AKO^) that is genetic deletion of *Esrra* in peripheral adipose tissues and most BMAds as previously reported,^29^ we established a spontaneous osteoarthritis and primary osteoporosis in male mice at the age of 25 months. We first determined metabolic cues in aged mice of the two genotypes and observed no significant differences in body weight, peritoneal WAT phenotype, and blood levels of total cholesterol (TC) and triglycerides (TG) (Fig. 1a and Fig. S1a, b). However, marrow fat accumulation induced by aging was substantially eliminated in the subchondral bone marrow and proximal metaphysis of tibia in *Esrra*^AKO^ mice in comparison to *Esrra*^fl/fl^ controls, as characterized by reduced positive immunofluorescence (IF) staining of perilipin 1 (PLIN1^+^), an adipocyte marker (Fig. 1b, c). Meanwhile, microCT analysis of proximal tibia revealed higher bone mass including trabecular bone volume per total volume (BV/TV) in aged *Esrra*^AKO^ mice compared to control littermates, as well as an increase in serum concentrations of procollagen type 1 N-terminal propeptide (P1NP, a bone formation marker) (Fig. S1c–e). Similar to our previous observations in estrogen deprivation-induced osteoporotic mice,^29^ these data suggest that loss of ESRRA in adipocytes dramatically impedes aging-induce MAT expansion while promoting bone formation.

To investigate whether adipocyte-specific abrogation of ESRRA affects osteoarthritis development, we first analyzed microCT imaging and histopathological changes of the knee joints in the two strains of mice, as previously described.^37–39^ As expected, 25-month-old *Esrra*^fl/fl^ mice were observed spontaneous osteoarthritis with sclerosis; whereas loss of *Esrra* resulted in a striking reduction in the volume of calcified meniscus and synovium, and thickening of the subchondral bone plate (SBP.Th). Compared to *Esrra*^fl/fl^ littermates, *Esrra*^AKO^ mice exhibited a significant decrease in the subchondral bone volume fraction (BV/TV) along with a lower trabecular bone pattern factor (Tb.Pf), indicating attenuated subchondral bone sclerosis (Fig. 1d-g and Fig. S1f). Consistently, manifestations of aging-induced osteoarthritis, namely cartilage degeneration lesion and synovial hyperplasia were substantially inhibited in *Esrra*^AKO^ mice, assessed by the Osteoarthritis Research Society International (OARSI) scores and synovitis scores (Fig. 1h–j). By IF staining, we next characterized that markers for catabolism (MMP13), DNA damage (γH2AX), senescence (P21) and apoptosis (TUNEL) and found that all of these parameters were downregulated significantly; whereas markers for chondrocyte anabolism collagen type II alpha 1 (COL2A1) and aggrecan (ACAN), and proliferation (Ki67) were enhanced in *Esrra*^AKO^ articular cartilage versus aged controls (Fig. 1k–n). Taken together, these data demonstrate that adipocyte ESRRA deficiency largely ameliorates aging-related spontaneous osteoarthritis, attenuating chondrocyte catabolism, senescence and apoptosis.

### Lack of *Esrra* in adipocytes attenuates pathogenesis of experimental osteoarthritis in mice fed with a HFHC diet

**Fig. 2:**
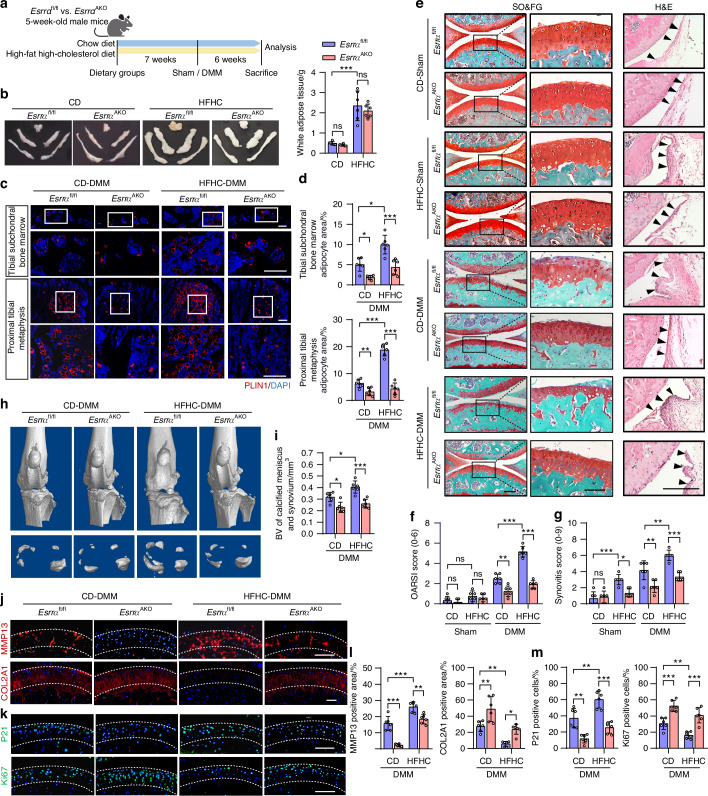
*Esrra* deficiency in adipocytes attenuates chondrocyte damage and experimental osteoarthritis pathogenesis induced by malnutrition and DMM. **a** Diagram illustrating the experimental osteoarthritis mouse model. Five-week-old *Esrra*^fl/fl^ or *Esrra*^AKO^ mice were fed either chow diet (CD) or high-fat high-cholesterol diet (HFHC) for 7 weeks, then they underwent medial meniscus destabilization (DMM) surgery on the right knee and sham surgery on the left knee, followed by 6 more weeks on their assigned diets before sacrifice. **b** Representative images and weights of WAT depots. **c**, **d** Immunofluorescence staining (**c**) and quantification (**d**) of PLIN1^+^ adipocytes area within the tibial subchondral bone marrow and proximal metaphysis from mice. Scale bar, 100 μm. **e** SO&FG-stained articular cartilage and H&E-stained synovium. Scale bar, 100 μm. **f**, **g** The corresponding quantitative data of OARSI score (**f**) and synovitis score (**g**). **h** Three-dimensional reconstruction of knee joint by microCT. **i** Quantitative analysis of the volume of the calcified meniscus and synovial tissue. **j**–**m** Immunofluorescence staining (**j**, **k**) and quantification (**l**, **m**) of MMP13, COL2A1, p21 and Ki67 in articular cartilage. Scale bar, 100 μm. For all experiments, *n* = 6 mice per group. Data are shown as mean ± SD. Statistical analysis is performed using two-way ANOVA with post-hoc Turkey’s multiple comparisons test

To determine whether the loss of *Esrra* in adipocytes protects against metabolic-related osteoarthritis, we established experimental osteoarthritis that is induced by an HFHC diet combined with DMM surgery as previously reported.^12^ Mice were fed either a long-term HFHC or normal chow diet (CD) for 13 weeks, and underwent DMM or sham surgery during the final 6 weeks of this period (Fig. 2a). As expected, HFHC feeding increased body weight, WAT mass and serum levels of TC and TG in comparison with CD-fed mice of both strains of mice, but no significant differences were observed between them (Fig. 2b and Fig. S2a, b). However, HFHC-fed *Esrra*^fl/fl^ mice exhibited profound osteopenia, which can be prevented by adipocyte *Esrra* abrogation (Fig. S2c–e). Consistent with a recent study,^31^ we also noticed that BMAds were accumulated in the proximal tibia and subchondral bone marrow in DMM mice which is comparable with that of HFHC-fed mice; and more accumulation of BMAds occurred in mice suffered the combined treatments (Fig. 2c, d and Fig. S2f, g). By contrast, ESRRA deficiency in adipocyte sufficiently inhibited marrow adiposity in these mice (Fig. 2c, d and Fig. S2f, g). By Safranin O and H&E staining as well as microCT imaging analysis, we determined that HFHC-fed mice exhibited exacerbated post-DMM osteoarthritis relative to CD-fed DMM controls, while deletion of adipocyte *Esrra* alleviated these osteoarthritis-like pathology (Fig. 2e–i and Fig. S2h, i). These evidence suggests that high marrow adiposity might be correlated with osteoarthritis development under diverse conditions. At molecular levels, we confirmed that MMP13, γH2AX, p21 and TUNEL induced by osteoarthritis were dramatically inhibited due to adipocyte ESRRA deficiency; meanwhile the loss of COL2A1, ACAN and Ki67 were preserved in the articular chondrocytes of *Esrra*^AKO^ mice versus corresponding controls (Fig. 2j-m and Fig. S2j–q). These results demonstrate that adipocyte-specific *Esrra* abrogation ameliorates the progression of metabolic-associated osteoarthritis, decelerating cellular senescence and catabolism in cartilage chondrocytes.

### Adipocyte ESRRA positively regulates the transcriptional expression of CFD by binding to its promoter

**Fig. 3:**
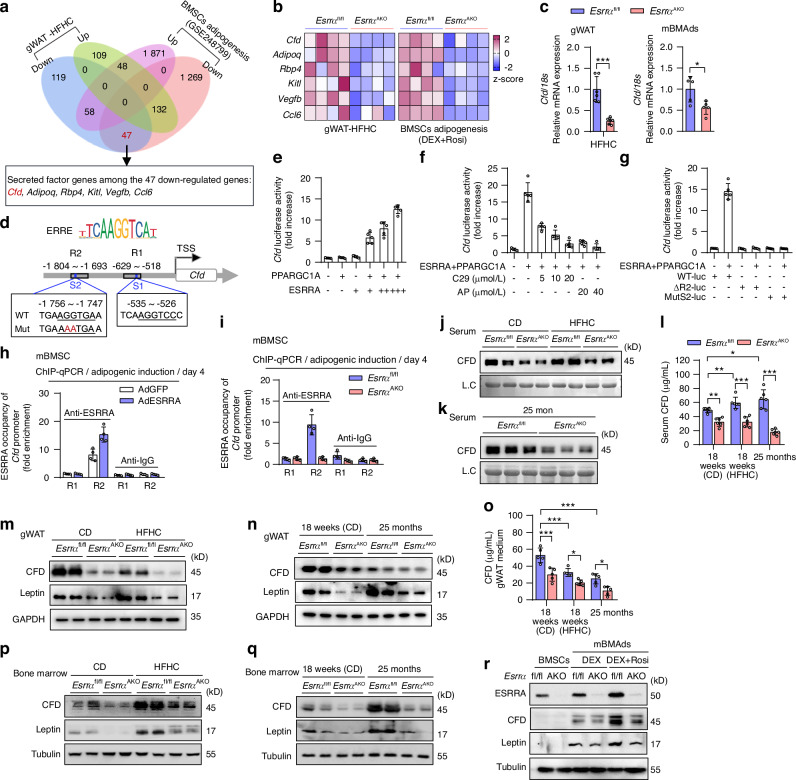
Adipocyte ESRRA positively regulates *Cfd* transcriptional expression responding to bone marrow adipocytes expansion. **a** Venn diagram of overlapping differentially expressed genes from RNA-seq of HFHC-fed gWAT and BMAds lineage cells in *Esrra*^fl/fl^ and *Esrra*^AKO^ mice (GSE248799), identifying six downregulated genes encoding secreted factors. **b** Heatmap of selected genes. **c** *Cf*d mRNA in HFHC-fed gWAT (*n* = 6) and fully differentiated mBMAds (*n* = 5). **d** Schematic of ERRE binding sites on the mouse *Cfd* promoter, with ChIP fragments indicated as region 1 and 2 (R1, R2). **e** Luciferase activity of *Cfd* promoter in 3T3‑L1 cells transfected with *Esrra* or *Ppargc1a* expression plasmids (*n* = 5). **f** Effects of compound 29 (C29) or andrographolide (AP) on ESRRA/PPARGC1A- driven *Cfd* promoter activity (*n* = 5). **g** Luciferase activities of the R2-deleted (ΔR2-luc), S2-mutated (MutS2-luc) and wild-type (WT-luc) *Cfd* promoter (*n* = 5). **h** ChIP assays with ESRRA antibody or IgG control in BMSCs after 4 days of adipogenic induction, with cells infected with ESRRA- or GFP-expressing adenovirus (*n* = 4). **i** Enrichment of ESRRA at *Cfd* promoter in BMSCs undergoing adipogenic induction from *Esrra*^fl/fl^ and *Esrra*^AKO^ mice (*n* = 4). **j**, **k** Immunoblots of serum CFD from HFHC-fed (**j**) and aged (**k**) groups versus control diet (CD). Ponceau S as loading control (L.C). **l** Serum CFD levels (*n* = 6). **m**, **n** Immunoblots of CFD and Leptin in gWAT from *Esrra*^fl/fl^ and *Esrra*^AKO^ mice as in (**j**, **k**). **o** CFD concentrations from culture medium of gWAT explants as in (**j**, **k**) (*n* = 5). **p**, **q** Immunoblots of bone marrow CFD as in (**j**, **k**). **r** Protein levels of ESRRA, CFD and Leptin in BMSCs and mBMAds with treatment of dexamethasone (DEX) with or without rosiglitazone (Rosi). Data are shown as mean ± SD. Statistical analysis is performed using two-sided unpaired Student’s *t*-test (**c**), two-way ANOVA with post-hoc Turkey’s multiple comparisons test (**l**, **o**)

The aforementioned data imply that ESRRA deficiency in adipocytes plays a key role in skeletal system through modulating certain secreted factors rather than disturbing systemic lipid metabolism. To delineate the functional role of ESRRA in adipocytes, we first carried out bulk RNA sequencing (RNA-seq) analysis in gonadal WAT (gWAT) from HFHC-fed mice. A total of 513 differentially expressed genes (DEGs) were identified (*P* < 0.05, average FPKM ≥ 10), of which 289 and 224 were respectively up- and down-regulated in gWAT from *Esrra*^AKO^ versus *Esrra*^fl/fl^ controls (Fig. 3a and Fig. S3a). Considering that high marrow adiposity induced by both aging and HFHC feeding was more responsive to suppression by ESRRA deficiency than peritoneal WAT, we therefore adopted our previously published RNA-seq dataset (GSE248799), which compares BMAd lineage cells from *Esrra*^AKO^ and *Esrra*^fl/fl^ mice.^29^ These BMAd lineage cells underwent adipogenic differentiation in vitro were concomitantly treated with dexamethasone (Dex) and rosiglitazone (Rosi) to mimic BMAds expansion responding to senescence or obesity.^29,40^ We combined these two datasets and identified 47 DEGs that were commonly downregulated in both gWAT and BMAd lineage cells from *Esrra*^AKO^, among which 6 candidate genes encoding *Cfd*, *Adipoq*, *Rbp4*, *Kitl*, *Vegfb* and *Ccl6* were recognized as secreted factors (Fig. 3a, b and Fig. S3b). Notably, CFD is a well-known adipokine, whose conventional role is to regulate complement activation that is involved in osteoarthritis development.^41^ Furthermore, MAT-derived CFD has been recently identified as a key factor promoting osteoporosis.^28^ qRT-PCR analysis also confirmed that *Cfd* mRNA expression was indeed inhibited robustly by *Esrra* ablation in gWAT of HFHC-fed mice as well as in fully differentiated BMAds (Fig. 3c). Thus, we propose that CFD might be a key functional modulator targeted by ESRRA in adipocytes.

Next, we ought to dissect whether ESRRA directly regulate the expression of *Cfd* by binding to two putative ESRRA response elements (ERREs) S1 and S2 on its corresponding promoter (Fig. 3d). We first confirmed that the regular *Cfd-*promoter is capable of responding to ESRRA with its co-activator PPARGC1A in a dose-dependent manner in 3T3-L1 preadipocytes after adipogenic differentiation for 2 days (Fig. 3e). By contrast, such transcriptional activities on the *Cfd*-promoter can be dose-dependently suppressed by two ESRRA antagonists compound 29 (C29) and andrographolide (AP), respectively^42,43^ (Fig. 3f). Moreover, region 2 truncation (ΔR2) and S2 mutation (MutS2) completely abolished ESRRA-mediated transactivation of the *Cfd*-promoter, demonstrating that ESRRA regulates *Cfd* transcription by binding to ERRE S2 but not S1 (Fig. 3g). We further evaluated if ESRRA is physically bound to ERREs on the *Cfd*-promoter by ChIP assays using primers encompassing regions R1 and R2 (Fig. 3d). Primary BMSCs were isolated and induced to undergo adipogenic differentiation for 4 days. ChIP-qPCR analysis confirmed the binding of endogenous ESRRA to R2 that contains S2, an occupancy that was further enriched by ESRRA overexpression (Fig. 3h). On the contrary, this binding was diminished in *Esrra*-ablated cells (Fig. 3i). A similar regulatory pattern was observed in differentiated 3T3-L1 cells following ESRRA transduction or treatment with C29 (Fig. S3c, d). Consistent with these observations, *Cfd* mRNA expression was increased in a dose-dependent manner by ESRRA overexpression in matured 3T3-L1 adipocytes and fully differentiated BMAds from murine or human BMSCs (Fig. S3e). Conversely, treatment of C29 or AP repressed *Cfd* expression (Fig. S3f). Together, these results reveal that *Cfd* is a bona fide ESRRA target gene in adipocytes.

### *Esrra* abrogation in adipocytes reduces circulating CFD levels that dominantly attributable to marrow adipocytes

To consolidate ESRRA-mediated CFD production and secretion in adipocytes in vivo, we validated that serum CFD levels were markedly reduced in *Esrra*^AKO^ mice as seen under aging and metabolic conditions, evidenced by either western blot or ELISA analysis (Fig. 3j–l). Noticeably, serum CFD levels were significantly enhanced by HFHC feeding and aging (Fig. 3j–l). However, CFD protein levels in gWAT were significantly diminished in HFHC-fed or aged mice (Fig. 3m, n). Accordingly, secretion levels of CFD were substantially reduced under the same conditions, as determined by explant culture of gWAT (Fig. 3o). A similar decrease was also observed in gWAT explants from female mice post-OVX (Fig. S3g). The suppressed production and secretion of CFD from expanded WAT was consistent with previous observations in several different obese mouse models, including *ob/ob* and *db/db* mice.^44,45^ These data suggest that a non-WAT source is responsible for elevated systemic CFD. Given that CFD has been characterized as a dominant adipokine released during MAT expansion in mice and humans,^28^ we propose that expanded MAT/BMAds, induced by HFD feeding and aging, are the primary contributor for elevated circulating CFD. Indeed, CFD levels in isolated bone marrow cells were increased in both aged mice and HFHC-fed mice, but remarkably reduced by *Esrra* ablation (Fig. 3p, q). Meanwhile, an adipocyte marker Leptin that is also a downstream target of ESRRA exhibited a parallel trend (Fig. 3p, q). Similar findings were observed when we analyzed bone marrow samples with expanded BMAds obtained from female mice eight weeks post-ovariectomy in our prior study^29^ (Fig. S3h). In agreement with these observations, fully differentiated BMAds with treatment of Rosi combined with or without Dex displayed incremented protein expression levels of CFD and Leptin, which were remarkably suppressed by *Esrra* knockout (Fig. 3r). Collectively, our data suggest that the expanded BMAds are the primary source of systemic CFD, while *Esrra* abrogation in adipocytes robustly inhibits CFD production and secretion particularly under conditions of pathological high marrow adiposity.

### *Esrra* ablation in BMAds counteracts mitochondrial dysfunction, cellular catabolism and senescence in chondrocytes via interrupting C3-CFD-MAC signaling-mediated ERK1/2 phosphorylation

**Fig. 4:**
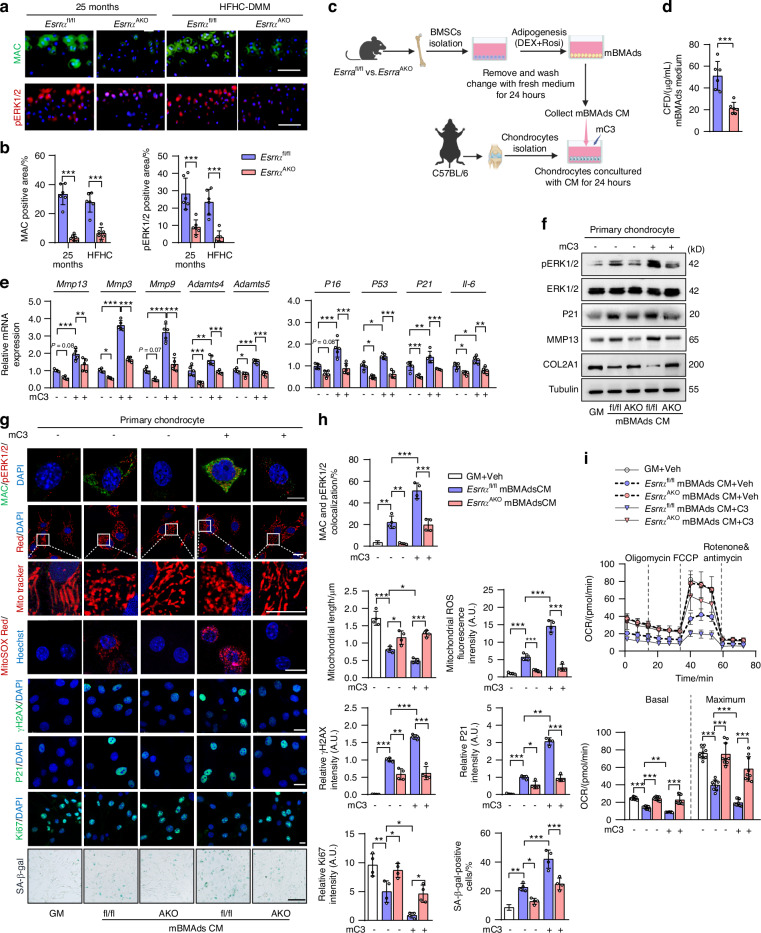
Reduced CFD from *Esrra*-ablated BMAds interrupts C3-CFD-MAC cascade-mediated mitochondrial dysfunction, senescence and catabolism in chondrocytes. **a**, **b** Representative images (**a**) and quantification (**b**) of MAC and pERK1/2 positive staining in cartilage regions of HFHC-fed or 25-month-old *Esrra*^fl/fl^ and *Esrra*^AKO^ mice. Scale bar, 100 μm. *n* = 6 mice. **c** Diagram depicting primary wild-type chondrocytes treated with the indicated conditioned medium (CM) collected from mBMAds, with or without supplementation of 1 μg/mL mouse recombinant C3 (mC3). **d** Soluble CFD concentrations from BMAds CM prepared as in (**c**) (*n* = 6). **e** mRNA levels of degradative enzymes *Mmp13*, *Mmp3*, *Mmp9*, *Adamts4*, *Adamts5* and senescent/proinflammatory markers *P16*, *P53*, *P21*, *IL6* in co-cultured primary chondrocytes as in (**c**) (*n* = 5). **f** Protein levels of pERK1/2, ERK1/2, P21, MMP13 and COL2A1 in co-cultured primary chondrocytes as in (**c**). **g**, **h** Immunofluorescence staining of MAC, pERK1/2, MitoTracker Red, MitoSOX Red, γH2AX, P21 and Ki67, as well as SA-β-gal staining in co-cultured primary chondrocytes (**g**). Quantitative analysis results are shown in (**h**) (*n* = 4). DAPI or Hoechst was used for nuclear counterstaining. Scale bar, 10 μm. **i** Oxygen Consumption Rate (OCR) were measured in co-cultured primary chondrocytes as in (**c**). Bar chart showing the results of mitochondrial basal and maximal respiratory capacity changes (*n* = 9). Data are shown as mean ± SD. Statistical analysis is performed using two-sided unpaired Student’s *t*-test (**b**, **d**), two-way ANOVA with post-hoc Turkey’s multiple comparisons test (**e**, **h**, **i**)

Considering that CFD as a rate-limiting enzyme plays a crucial role in the alternative complement pathway, contributing to the formation of the terminal effector MAC (C5b-9) on the cell membrane that can damage targeted cell,^46^ we assessed the osteoarthritic cartilages of both mouse models and observed that extensive MAC depositions were formed on the damaged chondrocytes in *Esrra*^fl/fl^ mice, which can be reversed by *Esrra* ablation in adipocytes (Fig. 4a, b). Moreover, we noticed enhanced extracellular signal-regulated kinase 1/2 phosphorylation (pERK1/2) together with MAC formation, which is consistent with a prior study in human osteoarthritic cartilage.^41^ To evaluate the effects of BMAd-derived CFD on chondrocytes at the cellular levels, we established a co-culture assay by primary chondrocytes derived from wild-type mice cultured with conditioned medium (CM) from BMAds treated with Rosi and Dex that mimic MAT expansion (Fig. 4c). We first confirmed that the concentrations of CFD released from *Esrra*^AKO^ BMAds were significantly lower than those from BMAds of *Esrra*^fl/fl^ (Fig. 4d). In primary chondrocytes treated with said CM and growth medium (GM) as the negative control, mRNA levels of degradative enzymes, *Mmp13*, *Mmp3*, *Mmp9*, *Adamts4* and *Adamts5*, were downregulated in *Esrra*^AKO^ CM groups, as well as senescent and proinflammatory markers, *P16*, *P53*, *P21* and *IL6* (Fig. 4e). Of note, the addition of recombinant mouse C3 (mC3) that acts as a substrate for CFD, exacerbated the detrimental effects of BMAds CM on chondrocytes, whose effects were largely attenuated in *Esrra*^AKO^ CM groups (Fig. 4e). Western blot analysis demonstrated that the phosphorylation of ERK1/2 was induced by *Esrra*^fl/fl^ BMAds CM and further exaggerated by mC3 challenge, which were substantially reversed in chondrocytes treated with CM from BMAds of *Esrra*^AKO^ mice (Fig. 4f). Consistently, the abnormal alterations in protein levels of P21, MMP13 and COL2A1 were greatly rectified in *Esrra*^AKO^ CM groups (Fig. 4f). These results demonstrate that ESRRA-mediated CFD release from BMAds is required for C3-innitiated alternative complement amplification loop in degenerated chondrocytes.

Since chondrocytes can undergo cellular senescence as a result of underlying cellular damage, we used confocal microscope to further explore the functional roles of CFD-mediated MAC in articular chondrocyte pathology. IF staining of MAC together with pERK1/2 colocalization showed abnormal distribution in chondrocytes treated with BMAds CM derived from *Esrra*^fl/fl^ mice, and displayed more deposits triggered by mC3 (Fig. 4g, h). By contrast, these colocalizations were markedly reduced following treatment with BMAds CM derived from *Esrra*^AKO^ mice (Fig. 4g, h). Mitochondria is a highly active organelle responding to metabolic stress, inflammation or aging, impairment of whose function aggravates oxidative stress and senescence. Since pERK1/2 induces mitochondrial fission and fragmentation by activating dynamin-related protein 1,^47^ we then assessed mitochondrial features and function. At the subcellular levels, we ascertained that combined treatment of BMAds CM from *Esrra*^fl/fl^ mice and mC3 compromised chondrocyte mitochondrion, as shown by the highest mitochondria-associated ROS levels and mitochondrial network fragmentation **(**Fig. 4g, h). By contrast, these mitochondrial defects were profoundly attenuated in chondrocytes treated with BMAds CM derived from *Esrra*^AKO^ mice, evidenced by MitoSOX Red and MitoTracker Red staining (Fig. 4g, h). Next, we evaluated the intensity of immunofluorescences of γH2AX, P21 and Ki67 located in the nucleus of chondrocytes, as well as β-gal staining. As expected, mC3 exacerbated chondrocyte senescence and damage upon treatment with BMAds CM from *Esrra*^fl/fl^ mice, which was rescued by the treatment of BMAds CM from *Esrra*^AKO^ mice (Fig. 4g, h). These data indicate that *Esrra* deficiency in adipocytes mitigates C3-CFD-MAC signaling-mediated mitochondrial dysfunction and senescence in chondrocytes via interfering the crosstalk between BMAds and chondrocytes.

Given that the anabolism of healthy chondrocytes relies on normal oxidative capacity, we further examined the mitochondrial oxygen consumption rate (OCR) in chondrocytes. Normal chondrocyte treated with control GM exhibited heightened states of OCR, which declined following treatment with BMAds CM (Fig. 4i). A further reduction in both basal and maximum OCR occured in chondrocytes exposed to combined BMAds CM from *Esrra*^fl/fl^ mice and mC3 (Fig. 4i). Conversely, the chondrocytes treated with BMAds CM from *Esrra*^AKO^ mice robustly restored mitochondrial respiration even upon mC3 challenge, indicative of preserved mitochondrial oxidative capacity and cellular anabolic capability (Fig. 4i). Taken together, these findings illustrate that reduced BMAds-derived CFD due to ESRRA deficiency ameliorates mitochondrial dysfunction, catabolic activity and senescence in chondrocytes via impeding the detrimental alternative complement activation and consequently excessive MAC-mediated pERK1/2 signaling.

### Steatotic liver contributes to elevated C3 in circulation across disparate metabolic-related skeletal disorders

**Fig. 5:**
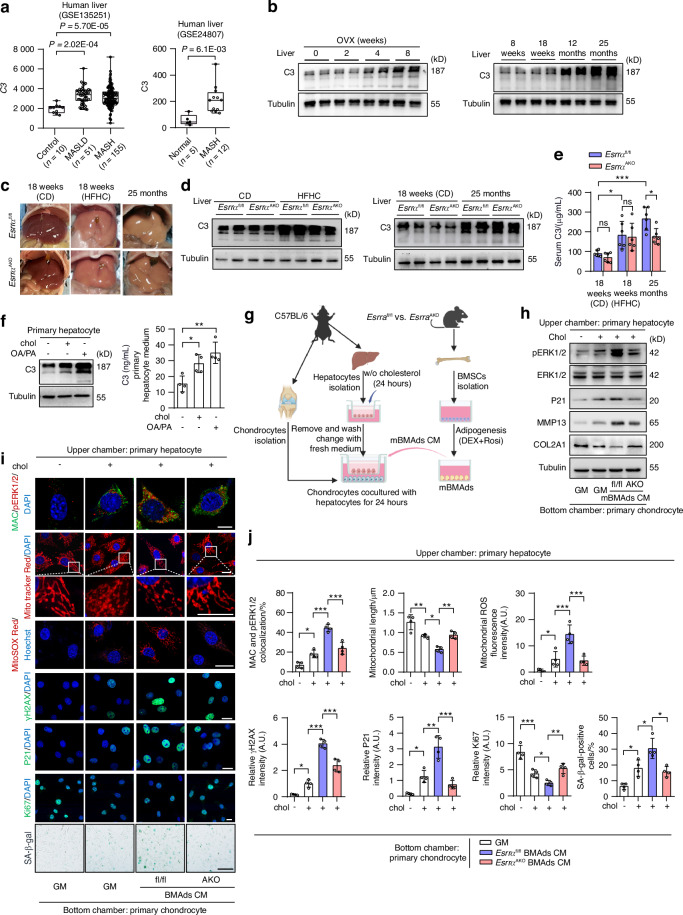
Inhibition of adipocyte ESRRA/CFD signaling rescues chondrocyte from damage caused by excessive hepatocytes-derived C3 under metabolic stress conditions. **a** Boxplots depicting C3 gene expression levels in MASLD/MASH patients compared to healthy controls from European (GSE135251) and American (GSE24807) cohorts. Data are represented as box and whiskers with bars representing maximum and minimum values and with median highlighted as a line. **b** Immunoblot analysis of C3 expression in livers from female mice at various time points following OVX (left) and male mice across different ages (right). **c** Representative liver images of the livers from HFHC-fed or 25-month-old *Esrra*^fl/fl^ and *Esrra*^AKO^ mice as well as corresponding controls. **d** Protein levels of C3 in the livers as in (**c**). **e** ELISA analysis of serum C3 concentrations. *n* = 6 mice per group. **f** The expression and secretion of C3 protein in primary hepatocytes following treatment with 40 μmol/L cholesterol (Chol) or a mixture of 250 μmol/L oleic acid (OA)/125 μmol/L palmitic acid (PA) for 24 hours (n = 4). **g** Diagram illustrating the co-culture system involving mBMAds CM, mouse primary hepatocytes and chondrocytes. **h** Western blot analysis of pERK1/2, ERK1/2, P21, MMP13 and COL2A1 in co-cultured primary chondrocytes as in (**g**). **i**, **j** Representative images (**i**) and quantitative analysis (**j**) of MAC, pERK1/2, MitoTracker Red, MitoSOX Red, γH2AX, P21, Ki67 and SA-β-gal staining in chondrocytes as in (**g**) (*n* = 4). DAPI or Hoechst was used for counterstaining of nuclei. Scale bar, 10 μm. Data are shown as mean ± SD. Statistical analysis is performed using two-sided unpaired Student’s *t*-test (right panel of **a**), two-way ANOVA with post-hoc Turkey’s multiple comparisons test (**e**, **j**), one-way ANOVA followed by Bonferroni’s post hoc tests (left panel of **a**, **f**)

The fine-balanced complement system is now appreciated as a system-wide modulator of biological processes, in which C3 ‘tickover’ is the initial trigger responsible for the amplification of alternative complement activity.^48^ Hepatocytes produce the majority of circulating C3, whose expression has been suggested to correlate with hepatic steatosis.^49–51^ To further support these evidences, we analyzed hepatic *C3* expression in two published transcriptome datasets (GSE 135251 and 24807) containing human liver samples from MASLD, MASH and corresponding controls. Indeed, a profound increase of C3 transcripts was presented in patients with MASLD/MASH (Fig. 5a). Based on these clinic observations, we speculate that excessive liver-derived C3 signaling contributes to the systemic skeletal comorbidities associated with metabolic stress. Thus, we employed female mice that underwent ovariectomy surgery, a model of estrogen deprivation that accelerates aging and increases susceptibility to osteoporosis, osteoarthritis, and hepatic steatosis; meanwhile we utilized wild-type male mice at different ages to represent natural aging (Fig. S4a, b). Western blot analysis revealed a gradual increase in C3 protein levels in these liver tissues along with the severity of steatosis (Fig. 5b, Fig. S4a, b). As expected, we observed augmented C3 levels in severely steatotic livers of both *Esrra*^fl/fl^ and *Esrra*^AKO^ mice induced by either aging or malnutrition, but no significant changes between the two strains of mice (Fig. 5c, d and Fig. S4c, d). Importantly, both HFHC feeding and aging resulted in a substantial increase in circulating C3 levels in the two strains of mice, compared to the corresponding young controls fed with a chow diet (Fig. 5e). Meanwhile, serum C3 levels tended to decline in aged *Esrra*^AKO^ mice compared to *Esrra*^fl/fl^ controls, implying that other sources might contribute to blood C3 contents in the context of aging (Fig. 5e). These data suggest a potential link between the excessive liver-derived C3 and skeletal disorders in diverse pathophysiological contexts including aging, malnutrition and estrogen deficiency.

### *Esrra* deficiency in BMAds mitigates chondrocytes damage caused by excessive C3 that released from lipid-overloaded hepatocytes

Having established the coexistence of increased levels of CFD and C3 resulting from high marrow adiposity and liver steatosis, we set out to determine whether hepatocytes-released C3 can serve as a substrate source driving alternative complement pathway involved in adipocyte-chondrocyte communication. To mimic metabolic stressed-liver caused by excess lipid accumulation, we challenged primary hepatocytes from wild-type mice with cholesterol or a mixture of free fatty acids combined oleic acid (OA) and palmitic acid (PA).^52^ We observed an apparent induction of C3 expression in hepatocytes treated with either cholesterol or fatty acids (Fig. 5f and Fig. S4e). Furthermore, augmented concentrations of C3 were detected in corresponding medium, revealing that excessive C3 can be produced and secreted from lipid-overloaded hepatocytes (Fig. 5f). Then we established a co-culture system in which hepatocytes treated with vehicle or cholesterol were seeded in the upper chamber, meanwhile primary wild-type chondrocytes were cultured in the bottom chamber. Additionally, the aforementioned conditioned medium of BMAds either from *Esrra*^AKO^ mice or from *Esrra*^fl/fl^ mice was added to this co-culture setup (Fig. 5g). Immunoblot analysis revealed that the chondrocytes co-cultured with cholesterol-treated hepatocytes exhibited increased expression of pERK1/2, P21 and MMP13 but decreased COL2A1 expression, which was aggravated by the additional treatment of BMAds CM from *Esrra*^fl/fl^ mice, suggesting that bona fide hepatocyte-derived C3-triggerd alternative complement pathway is amplified upon encountering adipocyte-secreted CFD (Fig. 5h). On the contrary, these alterations were markedly rescued by treatment of BMAds CM from *Esrra*^AKO^ mice (Fig. 5h). In accordance with these observations, we confirmed that treatment with BMAds CM from *Esrra*^AKO^ mice effectively blocked MAC deposits along with reduced pERK1/2 stimulation in chondrocytes co-cultured with cholesterol-treated hepatocytes (Fig. 5i, j). As expected, this blockade prevented chondrocytes from mitochondrial damage, cellular catabolism and senescence, as evidenced by IF staining and β-gal analysis (Fig. 5i, j). Collectively, these results indicate that *Esrra* deficiency in BMAds protects chondrocytes against the activation of C3-CFD-MAC pathway, particularly under conditions of aging- or metabolic disorders-related liver dysfunction.

### Inhibiting CFD by Danicopan or andrographolide impedes C3-triggered cellular senescence and catabolism in human chondrocytes

**Fig. 6:**
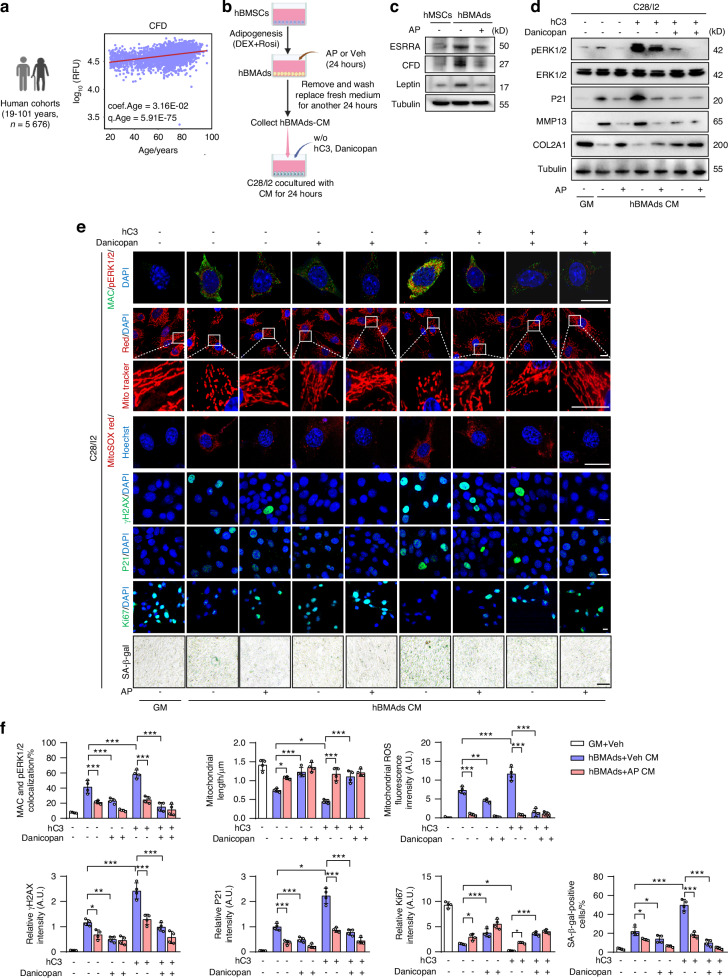
Suppressing CFD by Danicopan or andrographolide confers protection against cellular senescence and catabolism in human C28/I2 chondrocytes. **a** Scatter plot showing plasma CFD levels across the lifespan were analyzed using a public proteome dataset (see Method for details). RFU, relative fluorescent unit. **b** Illustration of the co-culture system involving C28/I2 chondrocytes and conditioned medium (CM) obtained from AP-treated human BMAds (hBMAds), with an additional treatment of 1 μg/mL human recombinant C3 (hC3) and/or 10 mmol/L Danicopan. **c** Western blot analysis of ESRRA, CFD and Leptin protein levels in human BMSCs-derived BMAds treated with 40 μmol/L AP or DMSO for 2 days. **d** Western blot analysis of pERK1/2, ERK1/2, P21, MMP13, and COL2A1 protein levels in human C28/I2 chondrocytes as in (**b**). **e**, **f** Representative images (**e**) and corresponding quantitative data (**f**) of MAC, pERK1/2, MitoTracker Red, MitoSOX Red, γH2AX, P21, Ki67 and SA-β-gal staining in human C28/I2 chondrocytes as in (**b**) (*n* = 4). DAPI or Hoechst was used for nuclear counterstaining. Scale bar, 10 μm. Data are shown as mean ± SD. Statistical analysis is performed using two-way ANOVA with post-hoc Turkey’s multiple comparisons test

Recently, two clinical cohort studies observe an increase in circulating CFD in aged humans, suggesting that blood CFD might be a biomarker of aging and aging-related diseases.^53,54^ Furthermore, postmenopausal women with low BMD also exhibited elevated levels of circulating CFD.^55^ Using plasma proteome data from independent human cohorts (https://twc-stanford.shinyapps.io/), we confirmed accentuated CFD levels with aging (Fig. 6a). Considering the biological distinctions between humans and rodents, we next evaluate the therapeutic potential of pharmacological inhibition of ESRRA/CFD signaling in modulating human adipocyte-chondrocyte communication (Fig. 6b). Human BMSCs (hBMSCs) were subjected to adipogenic differentiated using Dex and Rosi to model expanded and senescent hBMAds.^40^ To selectively inhibit human CFD activity, Danicopan, a US Food and Drug Administration (FDA)-approved oral drug, was introduced into the co-culture system alongside CM derived from these hBMAds. Western blot analysis proved that Danicopan treatment efficiently rectified the aberrant protein expression of pERK1/2, P21, MMP13 and COL2A1 in human chondrocyte cell line C28/I2 cells induced by hBMAds CM (Fig. S5a). Also, we employed AP as an ESRRA antagonist to suppress CFD expression in such senescent hBMAds, and subsequently collected the AP-treated hBMAds CM, together with control CM and growth medium (GM) as blank controls (Fig. 6b, c and Fig. S5b, c). The co-culture medium containing said CM from AP-treated hBMAds effectively mitigated damage in C28/I2 chondrocytes both with and without recombinant human C3 (hC3) supplementation, demonstrating that its protective effect is comparable to Danicopan treatment (Fig. 6d–f). Furthermore, combining Danicopan with AP-treated hBMAds CM in the co-culture medium markedly diminished hC3-triggered pERK1/2 expression as well as its co-localizations with deposited MAC in C28/I2 chondrocytes (Fig. 6d–f). These protective effects on mitochondrial defects, reduced cellular senescence and catabolism were evidenced by western blot and IF analysis (Fig. 6d–f). Taken together, these findings demonstrate that pharmacological inhibition of ESRRA/CFD signaling prevents chondrocyte damage by disrupting the alternative complement activation mediated by crosstalk between human BMAds and chondrocytes.

### Pharmacological inhibition of ESRRA by andrographolide protects against aging-related osteoarthritis in mice

**Fig. 7:**
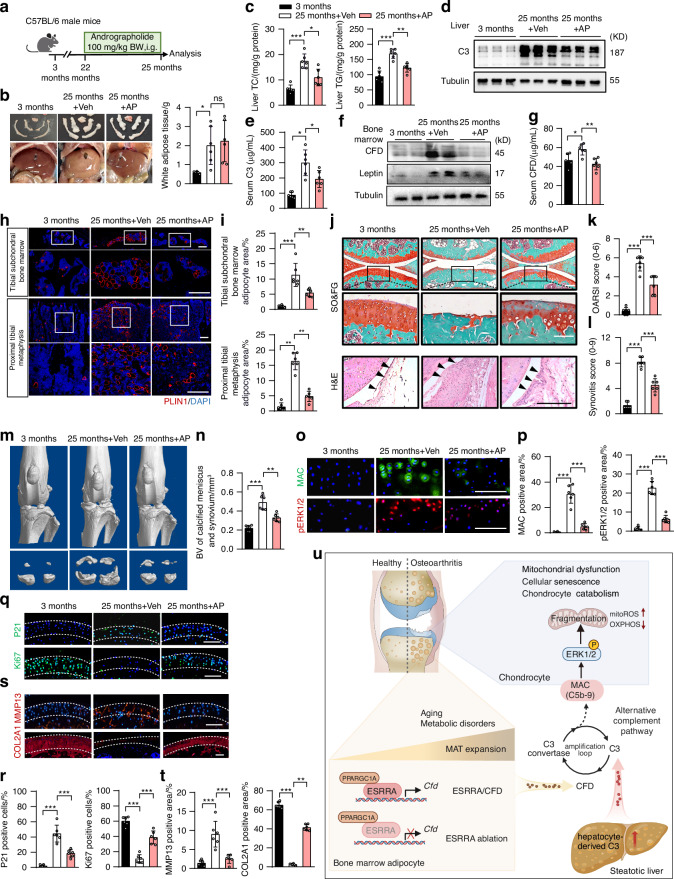
Pharmacological inhibition of ESRRA by andrographolide protects aged mice against spontaneous osteoarthritis progression. **a** Schematic diagram of the experimental design for pharmacological treatments in mice. 22-month-old male C57BL/6 mice were administered intragastrically (i.g.) with either vehicle or AP (100 mg/kg body weight) five times per week for 3 months. Young controls were 3-month-old male mice. **b** Representative images of WAT depots and livers, along with WAT weight analysis. **c** Liver TC and TG levels. **d** Immunoblots of C3 in liver tissues. **e** Serum C3 levels. **f** Immunoblots of bone marrow CFD and Leptin. **g** Serum CFD levels. **h**, **i** Immunofluorescence of PLIN1 staining (**h**) and quantification of PLIN1^+^ bone marrow adipocytes area (**i**). Scale bar, 100 μm. **j** Representative SO&FG-stained joint sections and H&E-stained synovial tissue. Scale bar, 100 μm. **k**, **l** OARSI score (**k**) and synovitis score (**l**). **m** Digital microCT images of knee joints. **n**, Quantitative data of the volume of calcified meniscus and synovial tissue. **o**, **p** Immunofluorescence staining for MAC and pERK1/2 in articular cartilage (**o**) and the quantitation (**p**). Scale bar, 100 μm. **q**–**t** Immunofluorescence staining (**q**, **r**) and quantification (**s**, **t**) of p21, Ki67, MMP13 and COL2A1. Scale bar, 100 μm. **u** A proposed model illustrates that ESRRA-mediated adipocyte CFD and liver-derived C3 synergistically promote osteoarthritis progression in aging and metabolic disorders. ESRRA transcriptionally upregulates CFD responding to MAT expansion. Together with steatotic liver-derived C3, this triggers excessive alternative complement activation, resulting in MAC deposition on chondrocytes, therefore provoking pERK1/2 activation and mitochondrial dysfunction. Targeting ESRRA alleviates osteoarthritis by interrupting this inter-organ C3-CFD-MAC cascade. For all experiments, *n* = 6 mice. Data are shown as mean ± SD. Statistical analysis is performed using two-way ANOVA with post-hoc Turkey’s multiple comparisons test

Lastly, we sought to examine the pharmacological effects of AP in preventing aging-related spontaneous osteoarthritis in vivo. 25-month-old wild-type C57BL/6 male mice were orally administered AP or vehicle (Veh) for three months prior to sacrifice (Fig. 7a). Compared to 3-month-old young male mice, aged mice exhibited elevated metabolic cues, including augmented body weight, visceral fat mass and serum levels of TC and TG, alongside severe MASLD marked by profoundly accumulation of hepatic TG and TC (Fig. 7b, c and Fig. S6a–c). In agreement with prior studies demonstrating the beneficial effects of AP on hepatic metabolism,^55^ oral administration of AP in aged mice reduced the circulating and hepatic levels of TG and TC as well as C3, alongside with alleviated histopathological features of MASLD but no significant changes in body weight and peritoneal WAT (Fig. 7b–e and Fig. S6a–d). CFD protein levels in isolated bone marrow were profoundly enhanced in aged mice compared to young mice; however, this increase was dramatically suppressed in AP-treated mice, indicating that AP functions efficiently as an ESRRA antagonist in vivo (Fig. 7f). Concurrently, elevated circulating levels of CFD in aged mice were completely restored in AP-treated aged groups (Fig. 7g). Consistently, we confirmed that AP treatment significantly reduced CFD expression and secretion in murine BMAds (Fig. S6e, f). Meanwhile, aging-induced MAT expansion was largely attenuated by AP treatment; and AP protected against aging-related osteoporosis as demonstrated by improved trabecular bone parameters and elevated P1NP levels (Fig. 7h, i and Fig. S6g–i). These data suggest that oral AP treatment in aged mice resulted in systemic suppression of C3/CFD signaling, counteracting high marrow adiposity.

Of note, the severity of aging-related spontaneous osteoarthritis was largely ameliorated in AP-treated aged mice, as demonstrated by substantial reductions in cartilage erosion, synovial hyperplasia and structural deterioration of knee joint (Fig. 7j-n and Fig. S6j, k). These observations coincided with decreased MAC-positive cells and inhibited pERK1/2 signaling in articular cartilage (Fig. 7o, p), as well as eliminated expressions of p16, MMP13, γH2AX and TUNEL (Fig. 7q–t and Fig. S6l–o). By contrast, augments in the expression levels of COL2A1, ACAN and Ki67 were observed in the articular chondrocytes of AP-treated aged mice as shown by IF staining, indicative of retention of the cartilage integrity (Fig. 7q–t and Fig. S6l–o). Taken together, these results reveal a potential therapeutic efficacy of AP for combating aging-related osteoarthritis in mice in part through inhibiting excessive activation of C3-CFD-MAC pathway.

## Discussion

Aging and metabolic disorders form a self-perpetuating vicious cycle accelerating skeletal deterioration. Herein, our findings suggest that excessive CFD from adipocyte-rich marrow contributes to osteoarthritis progression, bridging aging-related metabolic perturbations with steatotic liver, expanded MAT and degenerative cartilage through a C3-CFD-MAC signaling cascade. Mechanistically, we delineate that targeting ESRRA reduces the production and secretion of CFD from accumulated BMAds, by which interrupts the alternative complement activation triggered by hepatocyte-secreted C3, consequently blocking MAC-mediated pERK1/2 activation and mitochondrial dysfunction in cartilage chondrocytes (Fig. 7u). Our results reveal a previously unrecognized role of MAT and liver in regulating cartilage degeneration, providing novel insights into the inter-organelle crosstalk of alternative complement pathway that links metabolic and inflammatory regulation to age-related osteoarthritis (Fig. 7u).

Complement has long been considered a pivotal innate immune effector system, shaping tissue immunometabolism and homeostasis.^48^ The serum or synovial fluid levels of complement components elevate during osteoarthritis in patients, including C3, CFD and the terminal product MAC components that are C5, C7 and C9, indicating that alternative complement activation are associated with clinical outcomes.^22,41,56–58^ As a rate-limiting factor in the alternative complement pathway, CFD is produced and secreted almost exclusively by adipocytes.^44,45^ Expanded MAT can account for approximately 30% of total fat mass under pathological conditions and has been recently characterized as the dominant source for CFD in mice and humans during BMAds accumulation induced by aging, calorie restriction, fasting or thiazolidinedione treatment.^9,28,59^ We noticed that aging and HFHC feeding as well as estrogen deprivation result in profound BMAds accumulation in both epiphyseal and metaphyseal marrow in mice, associated with elevated circulation and bone marrow levels of CFD, reinforcing that CFD is dynamically regulated and most responsive factor to MAT expansion. Previously, we and others have dissected that adipokines leptin and CFD derived from accumulated BMAds dictate BMSCs fate toward adipogenesis, contributing to high marrow adiposity and bone loss, respectively.^28,29,60^ Notably, the serum concentrations of both leptin and CFD are positively correlated with osteoarthritis severity in patients.^22,56,61,62^ Moreover, genetic ablation of whole-body CFD in aged mice ameliorates spontaneous osteoarthritis and osteoporosis.^28,63^ We further demonstrate that inhibiting adipocyte CFD signaling confers protection against both conditions, suggesting that CFD not only serves as an aging biomarker but also represents a therapeutic target for degenerative skeletal disorders including osteoarthritis. These observations also raise the possibility that excessive CFD and bone-MAT imbalance may mutually exacerbate each other, propagating the comorbidity of osteoporosis and osteoarthritis. Moreover, HFHC exacerbates synovial hyperplasia in experimental osteoarthritis, aligning with studies demonstrating that high-fat diet aggravates synovitis in post-traumatic osteoarthritis.^64^ On the other hand, marrow adiposity like bone mass, exhibits sexual dimorphism.^65^ Notably, female mice possess lower bone mass and more marrow adipocytes in the proximal tibia than males, particularly following ovariectomy. Additionally, postmenopausal women and overiectomized rodents have high risk to develop osteoporotic osteoarthritis.^66–68^ Although the present study is primarily focused on male mice, our observations of liver steatosis and marrow adiposity in OVX mice suggest that dysregulated C3-CFD signaling, whether triggered by menopause or metabolic dysregulation, likely contributes to osteoarthritis development by increasing chondrocyte susceptibility to premature senescence.

As the substrate of CFD, C3 is mainly sourced from liver and essential for initiating the alternative complement activation cascade.^69^ The liver produces up to 90% of fluid-phase complement components, which typically circulate as plasma proteins and enter synovial fluid through filtration from the bloodstream.^70–72^ Although cartilage is an avascular tissue, the synovium undergoes neovascularization during synovitis which could facilitate complement proteins from the systemic circulation into the joint microenvironment.^72^ Emerging evidences show a positive correlation between serum C3 levels and MASLD in individuals.^49–51^ Moreover, this central complement component exhibits a linear correlation between its serum concentrations and synovial fluid.^71,73^ Of note, logistic regression analysis show that serum C3 is associated with the concomitant increased prevalence of cardiometabolic risk factors or MASLD in rheumatoid arthritis and spondyloarthritis patients.^74,75^ In current study, we observed that increased C3 abundance is produced and released from steatotic liver in mice when subjected to diverse pathophysiological conditions, including HFHC feeding, aging, and estrogen deprivation that modeling female menopause with accelerated aging features. When exposed to either cholesterol or OA/PA, primary hepatocytes exhibit a marked increase in C3 synthesis and secretion. Similarly, in human hepatocellular carcinoma Hep3B2 or HepG2 cell lines, C3 expression can be regulated by transcription factors C/EBP delta and hepatocyte nuclear factor 4 alpha (HNF4A), both of which are responsible for lipid and carbohydrate metabolism.^76,77^ These evidence suggests that increased hepatic C3 production and secretion induced by metabolic stress likely contributes to the development of osteoarthritis comorbid with MASLD/MASH.^17–19^ However, we cannot exclude the local sources of C3, CFD or other complement components in the regulation of inflammatory responses during osteoarthritis progression. The impact of systemic versus local complement modulations in osteoarthritis remains to be addressed. Importantly, emerging research highlights that liver-derived systemic factors can directly or indirectly drive the development of bone and joint diseases.^16,78–80^ Further work will be needed to characterize the functional role of liver-joint axis by hepatocyte-specific manipulation targeting C3 in vivo, particularly under metabolic disorder conditions.

Despite prior studies addressing the individual complement components in osteoarthritis,^41,62,63^ the functional role of the C3-CFD-MAC cascade has not been well studied in systemic regulatory contexts. Building on observed concomitant elevation of CFD and C3 resulting from high marrow adiposity and liver steatosis under aging-related metabolic conditions, we validate that hepatocyte-released C3 acts synergistically with BMAds-derived CFD to trigger and amplify the alternative complement pathway, driving extensive MAC deposition and pERK1/2 activation in cartilage chondrocytes. Consistently, MAC and pERK1/2 exhibit colocalization in human osteoarthritic cartilage associated with enhanced expression of proinflammatory and degradative enzymes.^41^ Our findings, using genetic models and co-culture system composed of multiple cell types, delineate that C3-CFD-MAC signaling-mediated pERK1/2 results in mitochondrial fragmentation and dysfunction, promoting cellular oxidative stress and chondrocyte senescence. This implicates that MAC as the terminal effector arm of C3-CFD complement cascade is a key driver of osteoarthritis.

With progressive aging, excessive cellular oxidative stress and damage due to mitochondrial dysfunction occur in the cartilage chondrocytes, contributing to the progression of osteoarthritis.^3^ Thus, an accumulation of complement factors such as C3 and CFD might be a pathological hallmark in aging-related osteoarthritis associated with metabolic disorders. In this regard, we propose that metabolic conditions and inflammatory activation may converge on a pathological C3-CFD-MAC cascade as a shared mechanism driving cartilage degeneration, reinforcing the intimate alternative complement regulation and interplay across tissues in aging-related metabolic osteoarthritis. Notably, osteoarthritis is recognized not merely as a cartilage disease but as a debilitating whole-joint disorder, in which microarchitectural alterations in subchondral bone may precede articular cartilage damage.^37,38^ In line with our observations, humans and mice with metabolic disorders exhibit a higher incidence of such osteoarthritis-related subchondral bone alterations than those without metabolic disorders.^37^ Importantly, adipocyte senescence propagates secondary senescence in the bone marrow; senescent cells such as preosteoclasts accumulate in subchondral bone and contribute to pathological alterations in HFD-challenged mice.^37,40^ Thus, it is of interest to investigate whether CFD inhibition in senescent BMAds protects against osteoarthritis progression by mitigating these pathological subchondral alterations in the context of metabolic stress or aging. On the other hand, we clarify that Danicopan effectively blocks hC3-triggered MAC deposition and damage in human chondrocytes. As a FDA-approved drug that selectively inhibits human CFD activity, Danicopan is of considerable interest to be investigated for osteoarthritis treatment in clinic.

Our mechanistic studies identify that CFD is a target gene of ESRRA. As a nuclear receptor, ESRRA regulates numerous genes in response to metabolic stress.^29,33^ CFD expression has been known to be transcriptionally regulated by another nuclear receptor peroxisome proliferator activated receptor gamma (PPARG), while strikingly repressed by PPARG deacetylation.^28,81^ We define that ESRRA deficiency markedly blunts CFD expression in senescent and expanded BMAds induced by co-treatment with dexamethasone and the PPARG agonist rosiglitazone, ascertaining ESRRA as indispensable for CFD production. A recent study identified CFD as a potential key downstream mediator of Leptin in adipose tissue, with both implicated in osteoarthritis and pain.^25^ Moreover, spatial transcriptomics reveal that CFD signal exclusively localizes within the bone marrow of the DMM limb in lipodystrophic mouse parabiotically joined to wild-type littermate.^25^ This observation suggests bone marrow CFD in the reintroduction of osteoarthritis in this parabiotic lipodystrophic mouse model.^25^ Given our prior demonstration that *Lep* is also a transcriptional target of ESRRA,^29^ pathological elevations of both CFD and Leptin are indeed effectively rectified by pharmacological inhibition and genetic ablation of *Esrra*, particularly in states of high marrow adiposity. While the precise in vivo contributions of reduced CFD and Leptin from specific adipose depots such as WAT versus MAT remain technically challenging to delineate, our collective data including BMAds analysis and conditioned medium assays strongly suggest that ESRRA inhibition in expanded BMAds plays an essential role in lowing circulating levels of these factors. Thus, reductions in these bone marrow-derived adipokines mediated by ESRRA inhibition likely functions in concert to mitigate the development of osteoarthritis.

Since the complement system is essential for homeostatic balance and health maintenance, targeting ESRRA may circumvent the infection risks caused by completely blocking the complement system.^33^ According to the adopt alternative conformation of ESRRA ligand-binding pockets that make its druggable, we and others previously characterized several synthetic and natural compounds as ESRRA inverse antagonists including C29 and andrographolide, oral administration of which ameliorate osteopenia or metabolic disorders in obese mice, respectively.^42,43^ Andrographolide has been commonly used in traditional Chinese and Indian medicines without any obvious side effects,^82,83^ thus we utilize it to inhibit ESRRA activity for osteoarthritis treatment. Oral administration of andrographolide in aged mice systemically confers protective effects against osteoarthritis and osteoporosis as well as MASLD, associated with declined levels of both C3 and CFD. We speculate that andrographolide might mitigate hepatic steatosis and therefore reduce C3 production in vivo via inhibiting hepatic nuclear factor 4 alpha activity.^75,84^ The observed impacts of andrographolide are of interest, implying that targeting multiple pathways might exert synergistic protection against aging-related skeletal diseases. Collectively, we demonstrate that targeting adipocyte ESRRA mitigates the pathogenesis of experimental and naturally occurring osteoarthritis though disrupting activation of the C3-CFD-MAC cascade mediated by a liver-MAT-cartilage axis. Our findings might broaden the understanding of systemic osteoarthritis pathobiology, highlighting the system-wide regulation of metabolism and immune function as therapeutic options of osteoarthritis.

## Methods

### Animal models

C57BL/6 mice were purchased from GemPharmatech (Nanjing, China). To generate adipocyte-specific Esrra knockout mice (AdipoqCre; *Esrra*^fl/fl^, refer as *Esrra*^AKO^), Adipoq-Cre mice were crossed with *Esrra*^fl/+^ mice as we previously described.^29^ In this study, all mice had free access to water and were maintained under a 12-hour light-dark cycle. They were housed in pathogen-free barrier facilities at a constant temperature of (24 ± 2) °C and humidity of 60% ± 5%. All procedures involving the animals and their welfare were thoroughly reviewed and subsequently approved by the Ethical Review Board of the Shenzhen Institutes of Advanced Technology, Chinese Academy of Sciences.

For aging-induced osteoarthritis in mice, *Esrra*^fl/fl^ and *Esrra*^AKO^ mice were raised under standard conditions until 25 months of age. To establish the experimental osteoarthritis model involved metabolic and mechanical factors, mice received a high-fat high-cholesterol diet (HFHC) combined with DMM surgery as previously described.^12^ In Brief, 5-week-old *Esrra*^fl/fl^ or *Esrra*^AKO^ mice were assigned to either a control chow diet (CD) or an HFHC diet (Research Diets, #D09100310) containing 40 kcal% fat (primarily palm oil) and 2% cholesterol. After 7 weeks, mice underwent DMM surgery on the right knee and sham surgery on the left knee, and remained on assigned diets for 6 weeks before analysis. For andrographolide (AP, Sigma #365645) treatment experiments, 22-month-old male C57BL/6 mice were administrated daily oral gavage **(**5 days per week) of either vehicle control (40% corn oil, 6% vitamin E-TPGS, 12% PEG400, and 42% water) or AP (100 mg/kg body weight) for 3 months until age 25 months. Male C57BL/6 mice at 3 months of age served as young controls. All mice were fasted overnight prior to being anesthetized by isoflurane inhalation and euthanized by cervical dislocation. Knee joint, bone, white adipose tissue, serum, and liver samples were collected for analysis. Serum triglycerides (TG, Applygen #E1003) and total cholesterol (TC, Applygen #1005) levels were measured according to the manufacturer’s protocol.

### Micro-computed tomography (CT) analysis

For the analysis of osteoarthritis and bone mass, knee joints and tibiae were dissected to remove soft tissues and subsequently fixed in 4% paraformaldehyde (PFA) (Beytime #P0099). The samples were prepared for microCT scanning (SCANCO MEDICAL #µCT 100) with the following parameters: 50 kVp voltage, 450 μA current, and a resolution of 9 µm per pixel. The joint and trabecular bone structure were reconstructed using NRecon software. Following reconstruction, the scans were aligned in the same plane using DataViewer. Image analysis was performed using CTAn software, and three-dimensional images were generated using CTvol software. Subchondral bone structural parameters analyzed on the medial tibial plateaus included subchondral bone plate thickness (SCBP), tibial subchondral bone volume fraction (BV/TV), and trabecular bone pattern factor (Tb.Pf).

### Histological preparation and histological evaluation

Mice whole knee joints and the attached tibiae were fixed in 4% paraformaldehyde (PFA) and subsequently decalcified in 0.5 mol/L EDTA (pH 7.2) at 4 °C for up to 4 weeks. The specimens were then embedded in paraffin, sectioned into 5 μm slices. The sections were dewaxed and rehydrated with xylene and gradient alcohol. Subsequently, staining procedures were carried out using either the Safranin O/Fast Green (SO&FG) staining kit (Solarbio #G1371) or hematoxylin and eosin (H&E) kit (Beyotime #C0105S). The fluorescent signals in the articular cartilage regions were quantified using a standard microscope (Olympus BX53). Histological scoring, quantitative histology staining analyses were conducted in a double-blind manner.

### Immunofluorescence staining

Paraffin sections were prepared following previously established procedures. Following deparaffinization and rehydration, antigen retrieval was carried out in 1% Proteinase K for 6 minutes or 0.1% Trypsin for 25 minutes at 37 °C. Sections were permeabilized and blocked with 1% donkey serum and 0.1% Tween 20 (Sigma # 9005-64-5) and 0.1% Triton X-100 (Sigma #9036-19-5) for 0.5 hour and incubated with primary antibodies overnight at 4 °C. The primary antibodies used included COL2A1 (1:200, Proteintech #28459-1-AP), Aggrecan (1:200, Proteintech #13880-1-AP), MMP13 (1:200, Proteintech #18165-1-AP), P21 (1:200, Proteintech #28248-1-AP), Ki67 (1:200, Proteintech #28248-1-AP), γH2AX (1:200, Proteintech #28248-1-AP), pERK1/2 (1:200, Selleck #F1716), Perilipin-1 (1:200, Cell Signaling Technology #9349) and C9 (1:200, Novus Biologicals #NBP2-15952). Immunofluorescence staining with the C9 antibody was performed to visualize MAC deposition. Sections were incubated with secondary antibodies, Goat Anti-Rabbit IgG H&L (Alexa Fluor® 488) (Abcam #ab150077) or Goat Anti-Rabbit IgG H&L (Alexa Fluor 555) (Abcam #ab150078). Nuclei were counterstained with DAPI (Cell Signaling Technology #4083). After two washes with PBS, sections were dried and mounted with an anti-fade reagent (Invitrogen #P10144). For TdT-mediated dUTPNick-EndLabeling (TUNEL) staining, TUNEL BrightGreen Apoptosis Detection Kit (Vazyme #A112) was used according to the manual of manufacturer’s instructions. The images were captured with Olympus light microscope. Cartilage-positive areas were quantified using ImageJ software. The proportion of positive cells was calculated as the percentage of positive cells relative to the total number of DAPI-stained nuclei.

### Isolation and adipogenic differentiation of BMSCs

As described, adipocytes were differentiated from bone marrow stromal/stem cells (BMSCs).^29^ Briefly, primary mouse BMSCs were isolated from the bone marrow. BMSCs were differentiated using 5 ng/mL insulin (Yeasen #40112ES25), 0.5 mol/L IBMX (Sigma #I5879), and 1 μmol/L dexamethasone (DEX) (Sigma #D4902) in α-MEM (Gibco #C12571500BT) containing 10% FBS (TransGen Biotech #FS301) for another two days, with or without 1 nmol/L rosiglitazone (Sigma #R2408), and then were only treated with insulin for two days. When specified, fully differentiated BMAds were treated with DMSO, 20 or 40 μmol/L andrographolide for 2 days. Alternatively, differentiated BMAds were infected with adenovirus expressing ESRRA (50 pfu or 100 pfu) or GFP for two days. Adenoviruses were produced and purified as we described previously.^29^ Subsequently, BMAds conditioned medium was collected for coculture experiments. Adipocytes were identified by Oil Red O staining (Solarbio #G1262). The supernatants were collected as BMAds conditioned medium (BMAds-CM) and used for further experiments.

Human fetal BMSCs (hBMSCs) were purchased from Cyagen Biosciences Inc. (#HUXMF-01001). The immunophenotype of the purified stromal cell population was characterized by flow cytometry analysis using specific surface marker proteins CD44, CD29, CD105, CD34, and CD45. The maintenance and adipogenic differentiation of hBMSCs, as well as the collection of BMAds CM, were carried out following the same procedures described for mouse BMSCs. Only cells that had undergone three passages or fewer were used for in vitro experiments.

### ELISA assay

ELISA assays were performed to detect CFD (R&D Systems #DY5430-05), C3 (Cloud-clone #SEA861Mu), and P1NP (Novus Biologicals #NBP2-76466). For CFD secretion analysis, gWAT were freshly isolated from mice and subjected to explant culture. Briefly, gWAT explants were finely mincing into pieces of approximately 20 mg fragments. The minced tissue were cultured in α-DMEM containing 10% FBS at 37 °C for 48 hours. The culture supernatant was collected and further analyzed. All ELISA procedures were conducted in accordance with the manufacturer’s protocol.

### Primary chondrocyte isolation

The neonatal C57BL/6 mice were disinfected with 75% alcohol. A 2 mm incision was made on the knee of the neonatal mice. The cartilage was dissected and minced in a sterile environment. After digestion with 0.4% type II collagenase (Gibico #17101015) at 37 °C for 3 hours, the mixture was centrifuged at 1 000 r/min for 4 minutes. The cell suspension was then filtered through a 70-micron filter to isolate chondrocytes. Primary chondrocytes were cultured in DMEM/F12 medium (Gibico #C11330500BT) with 10% FBS and 1% penicillin/streptomycin (Hyclone #SV30010). The passage 2 (P2) generation cells were used for the experiments.

### Primary hepatocyte isolation

To isolate primary hepatocytes, a two-step collagenase perfusion method was employed. Following digestion with collagenase type I (Sigma, V900892), the liver tissue of C57BL/6 mouse was minced, filtered through a 70-μm cell strainer, and centrifuged to collect hepatocytes. Murine hepatocytes were isolated by low-density centrifugation with Percoll (Bioregen #BN31708). Hepatocytes viability exceeded 90% as assessed by trypan blue exclusion. After being plated, the attached cells were nurtured overnight in low-glucose DMEM (Hyclone #SH30021.01) supplemented with 10% FBS, 1% penicillin-streptomycin, and 10 mmol/L HEPES (Solarbio #H1095).

### Chondrocyte coculture assay

Primary hepatocytes were seeded into type-I collagen-coated upper chambers of Transwell inserts (Corning #354236/#3412). The hepatocytes were pre-treated for 24 hours with either 40 μmol/L soluble cholesterol (Sigma #C4951), or a mixture of 250 μmol/L oleic acid (Sigma #O1383) and 125 μmol/L palmitic acid (Kunchuang Biotechnology #KC003). Subsequently, hepatocytes were co-cultured with primary chondrocytes in the bottom chamber for an additional 24 h in medium supplemented with indicated mBMAds CM or control growth medium (GM). The human chondrocyte cell line C28/I2 (Shanghai Cell Bank, China) was cultured in DMEM medium containing 10% FBS, then exposed to the indicated human BMAds CM or control GM. In specified experiments, culture medium was supplemented with 1 μg/mL mouse recombinant C3 (mC3) (Cloud-clone #RPA861Mu01), or 1 μg/mL human recombinant C3 (hC3) (Cloud-clone #RPA861Hu01) with or without 10 μmol/L Danicopan (synonym: ACH-4471) (MCE #HY-117930), and incubated for 24 h.

### Cell immunofluorescence

Cells were then washed with PBS and fixed with 4% PFA for 15 minutes. Permeabilization was performed with 0.1% Triton-X (Sigma #T8787) for 5 minutes. The cells were blocked for with 1% BSA (Yeason #36104ES25) for 1 hour, followed by overnight incubation with the primary antibodies at 4 °C. The antibodies used for analysis included Ki67 (1:200, Huabio #HA721115), γH2AX, pERK1/2 (1:200, Selleck #F1716), mouse MAC (C5b-9) (1:50, Santa Cruz #sc-66190 AF488), and human MAC (C5b-9) (1:50, Santa Cruz #sc-58935). Secondary antibodies were applied for 1 h at room temperature. Nuclei were stained with DAPI for 20 min. After washing with PBS, the slices were sealed with an anti-fade fluorescence mounting medium. Fluorescence was observed using confocal microscopy (Leica STELLARIS 5).

### MitoTracker Red and MitoSOX Red staining

For mitochondrial morphology analysis, live cells were stained with MitoTracker Red (200 nmol/L, Invitrogen #M22425) at 37 °C for 30 min. The cells were then fixed with pre-chilled methanol for 15 minutes. After DAPI staining, coverslips were sealed, and observations were made using confocal microscopy. For mitochondrial-derived ROS, live cells were washed with PBS, incubate with MitoSOX Red working solution (5 μmol/L, Invitrogen #M36008) at 37 °C in the dark for 10 min. Cell-permeable DNA dye Hoechst 33342 (1:1 000, Beyotime #C1029) was added to the incubation plate according to the manufacturer’s direction. Observations were made using confocal microscopy.

### β-galactosidase staining

Fixed cells were stained with β-galactosidase (β-gal) dye for 48 h using the β-gal Staining Kit (Solarbio #G1580). After washing three times with PBS, the stained cells were observed under a phase-contrast microscope in PBS to determine the percentage of positive cells relative to the total cell population.

### Seahorse extracellular flux analysis

Oxygen consumption rate (OCR) values were measured for 30 000 cells per well using the Agilent Seahorse XF Analyzer XF96. Baseline OCR measurements were obtained to establish a reference point for subsequent analyses. Oligomycin (1.5 μmol/L), an ATP synthase inhibitor, was then added to assess ATP-linked oxygen consumption. The OCR values after oligomycin addition were recorded to determine the extent of ATP-linked respiration. To evaluate maximal respiratory capacity, FCCP (1 μmol/L), a protonophore that uncouples oxidative phosphorylation, was subsequently added. Finally, rotenone (0.5 μmol/L) and antimycin A (0.5 μmol/L), inhibitors of complexes I and III of the electron transport chain, were introduced to measure non-mitochondrial oxygen consumption. The final OCR value was calculated by subtracting the OCR measured after rotenone and antimycin A treatment from the OCR measured after FCCP addition.

### RNA extraction and real-time qPCR

Total RNA was extracted from tissues or cultured cells using AG RNAex Pro Reagent (Accurate Biotechnology #AG21101). cDNA was synthesized by reverse transcription using reagents from Takara (#RR037). Real-time PCR was performed on a qTOWER3 real-time PCR system (Analytik Jena) using the Real-Time PCR Mix (GenStar #A301-10) and specific primers (Supplementary Table S1). The relative mRNA expression levels were quantified using the 2^−ΔΔCt method. The 18S rRNA gene was used as an endogenous control to normalize the total RNA input. All experiments were conducted in at least four biologically independent replicates.

### Western blot analysis

Cells and tissues were lysed in RIPA buffer (Beyotime #P0013B) supplemented with a protease inhibitor cocktail (MCE #HY-K0010) and phosphatase inhibitors (Selleck #B15001). The resulting cell lysates were mixed with SDS loading buffer (Beyotime #P0015L) and subjected to electrophoresis. Proteins were then transferred onto a 0.22 μm PVDF membrane (Merck Millipore #IPFL00010). For serum analysis, the band intensities in Western blots were normalized to those obtained by Ponceau S staining (Beyotime #P0022). Membranes were blocked with 5% nonfat milk (Sangon Biotech #A600669) and incubated with primary antibodies overnight at 4 °C, followed by incubation with appropriate secondary antibodies for 2 h. Immunoreactive proteins were visualized using the ECL luminescence reagent (Millipore #WBLUR0500). The antibodies used for immunoblot analysis included ESRRA (1:1 000, Cell Signaling Technology #13826), Tubulin (1:5 000, BPI #AbM59005-37B-PU), mouse CFD (1:800, R&D Systems #AF5430), human CFD (ABclonal #A23006), C3 (1:5 000, ABclonal #A13283), GAPDH (1:5 000, Proteintech #60004-1-Ig), COL2A1 (1:800, Proteintech #28459-1-AP), MMP13 (1:1 000, Proteintech #18165-1-AP), P21 (1:1 000, Proteintech #28248-1-AP), pERK1/2 (1:2 000, Abmart #T40072S), ERK1/2 (1:2 000, Abmart #T40071S), and Leptin (1:500, Abcam #ab16227). The immunocomplexes were incubated with corresponding secondary antibodies: HRP-goat anti-mouse IgG (1:4 000, EarthOx Life #E030110-02), HRP-goat anti-rabbit IgG (1:4 000, EarthOx Life #E030120-02), or HRP-rabbit anti-sheep IgG (1:1 000, EarthOx Life #E030150-01). Images of the samples were captured using the ChemiDoc XRS chemiluminescence imaging system (Bio-Rad).

### RNA-Sequencing data processing and analysis

For RNA extraction, gonadal adipose tissue was homogenized and lysed with TRIzol reagent. Following phase separation, the aqueous phase was transferred to spin columns from the PureLink RNA Mini Kit (Vazyme #RC101-01) and processed according to the manufacturer’s instructions. Subsequent cDNA library was constructed using TruSeq RNA Sample Prep Kit and then sequenced on HiSeq 2000 system at Shanghai Biotechnology Corporation (Shanghai, China). For data analysis, sequence read alignment was conducted using the STAR aligner, and fragments per kilobase million (FPKM) quantification was performed using RSEM. Differential expression analysis was conducted using two-sample *t*-tests. Genes with a *P*-value < 0.05 and an average FPKM ≥ 10 were considered significantly differentially expressed. Venn diagram analysis was performed to identify commonly differentially expressed genes by comparing the RNA-seq dataset of MSC adipogenic differentiation over 7 days in the presence of Dex and Rosi with the RNA-seq results from gWAT. Subsequently, secretory proteins were analyzed and predicted using the Secreted Protein Database (SEPDB, available online at https://sysomics.com/SEPDB/) to identify differentially expressed genes encoding secretory proteins.

### Plasmid construction and luciferase reporter assays

The regulation of ESRRA on *Cfd* transcription was evaluated by dual luciferase reporter assay. Luciferase reporter vectors containing the wild-type, truncated and mutated forms of the *Cfd* promoter were constructed in the pGL3-Basic plasmid using specific primers (Supplementary Table S2). Subsequently, the *Cfd* promoter plasmid was co-transfected with *Esrra*, *Ppargc1a*, and pRL-TK plasmids into 3T3-L1 cells using Lipofectamine 3000 transfection reagent (Invitrogen#L3000015), with or without the addition of an ESRRA inverse agonist Compound 29 (C29) (ChemPartner #S1039) or AP. After 24 hours, the cells were lysed, and luciferase activity was measured with a dual-luciferase reporter assay system (Promega, E1960) and Microplate Luminometer (Promega Glo-Max 96, E6521). The final analysis was normalized to Renilla luciferase activity.

### Chromatin immunoprecipitation (ChIP) assay

ChIP was performed essentially as we previously described.^29^ Briefly, primary BMSCs and 3T3-L1 preadipocytes were harvested 4 days after the induction of adipocyte differentiation. Then, cells were cross-linked for 10 min in 1% formaldehyde in PBS, followed by quenching with 0.125 mol/L glycine. After the chromatin was sheared on an Ultrasonic Processor (Diagenode), immunoprecipitation was carried out using antibodies to ESRRA (10 μL/IP) and Rabbit IgG (1 μg/LP, Cell Signaling Technology #2729). After pull-down with Protein G magnetic beads (Cell Signaling Technology #9006), RNA and protein digestion, DNA purification was conducted in accordance with the manufacturer’s instructions (Cell Signaling Technology #9005). The enrichment of ESRRA in the *Cfd* promoter region was quantified using RT-qPCR with specific primers (Supplementary Table S3). The quantity of immunoprecipitated DNA in each sample was normalized to IgG-associated DNA, thereby determining the relative fold enrichment.

### Public datasets

A variety of omics resources were incorporated into this study. An online platform developed for analyzing plasma protein expression across a broad age spectrum in a cohort of 5 676 individuals at the age of 19-101 years was utilized (https://twc-stanford.shinyapps.io/aging_plasma_proteome_v2/). The CFD protein was assessed by quantifying its q-value and age coefficient. Additionally, the publicly accessible datasets and databases employed in this study include the following: RNA-seq data from primary BMSCs underwent adipogenic differentiation for 7 days with treatment of dexamethasone and rosiglitazone in our prior study (GEO accession: GSE248799)^29^; human liver RNA-seq data sourced from two repositories (GEO accessions: GSE135251 and GSE24807).

### Statistical analysis

Systematic allocation and not randomization was used to distribute animals to experimental groups. Normal distribution was assumed. Statistical differences across more than two groups were assessed using two-way ANOVA with post-hoc Turkey’s multiple comparisons test or one-way ANOVA followed by Bonferroni’s post hoc tests as detailed in the figure legends. Unpaired, two-tailed Student’s *t*-test was employed for two-group comparison. Data are expressed as mean ± SD. A *P*-value < 0.05 was considered statistically significant. Statistical analyses were conducted using GraphPad Prism 8.0.

## Supplementary information

Supplementary information

## Data Availability

All data associated with this study are included in this article or its supplementary materials. The bulk RNA-seq data from gWAT of HFHC-fed *Esrra*^fl/fl^ and *Esrra*^AKO^ mice have been deposited in the GEO database under accession code GSE301065.
