# Supplementary material for: Targeting adipocyte ESRRA alleviates osteoarthritis via interrupting inter-organelle crosstalk of complement C3-CFD-MAC cascade
Source: Bone Res. 2026 Apr 29;14:49. doi: 10.1038/s41413-026-00527-3 (PMC13128952; doi:10.1038/s41413-026-00527-3)
Supplement: Supplementary file 1 — Supplementary information [file 41413_2026_527_MOESM1_ESM.docx]

**Supplementary information for:**

**Targeting adipocyte ESRRA alleviates osteoarthritis via interrupting inter-organelle crosstalk of complement C3-CFD-MAC cascade**

Tongling Huang, Zihui Wang, Lu Gao, Jun Gao, Zhaocheng Lu, Pengda Li, Chon Him Choy, Zhuolei Yuan, Yanting Zhong, Chang-An Geng, Huaiyu Wang, Kelvin W K Yeung, Bin li, Haobo Pan, Di Chen, Min Guan*

***Correspondence:** Min Guan, Ph.D. Institute of Biomedicine and Biotechnology, Shenzhen Institutes of Advanced Technology, Chinese Academy of Sciences.

1. **mail:** min.guan@siat.ac.cn

**Supplementary information contents:**

1. Supplementary Figures: 1-6

2. Supplementary Tables: 1-3


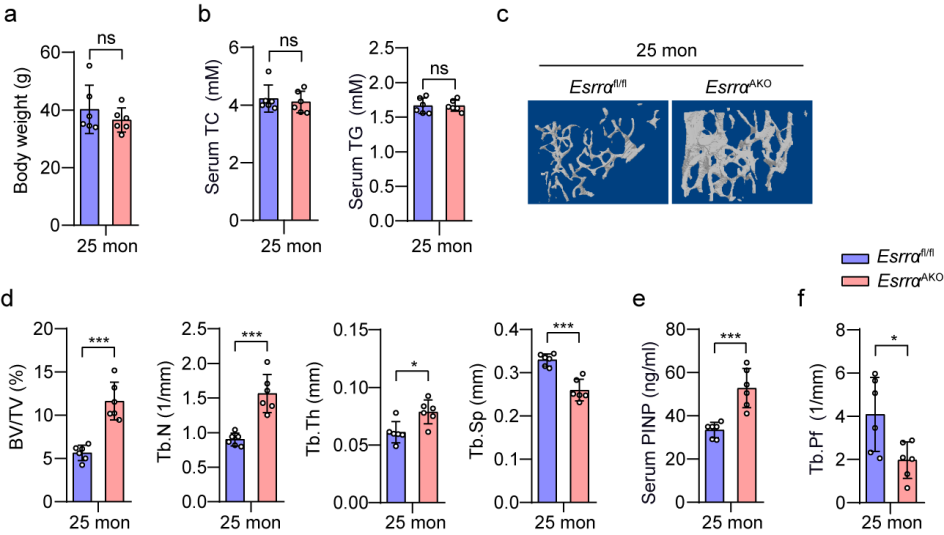


**Supplementary Fig. 1 Adipocyte *Esrra* ablation results in no significant changes in body weights and blood biochemistry, while enhances bone mass in 25-month-old mice.**

**a**, Body weights analysis of 25-month-old *Esrra*^fl/fl^ and *Esrra*^AKO^ mice. **b**, Serum total cholesterol (TC) and triglyceride (TG) levels. **c**, Three-dimensional reconstruction from micro-CT scans of tibial trabecular bone. **d**, Bone histomorphometric analysis of bone volume/tissue volume ratio (BV/TV), trabecular number (Tb. N), trabecular thickness (Tb. Th) and trabecular separation (Tb. Sp). **e**, Serum P1NP levels. For all experiments, n = 6 mice per group. **f**, Quantitative analysis of trabecular pattern factor (Tb.Pf) in the medial side of tibial subchondral bone of 25-month-old *Esrra*^fl/fl^ and *Esrra*^AKO^ mice. Data are shown as mean ± SD. Statistical analysis is performed using unpaired two-tailed Student's *t*-test.


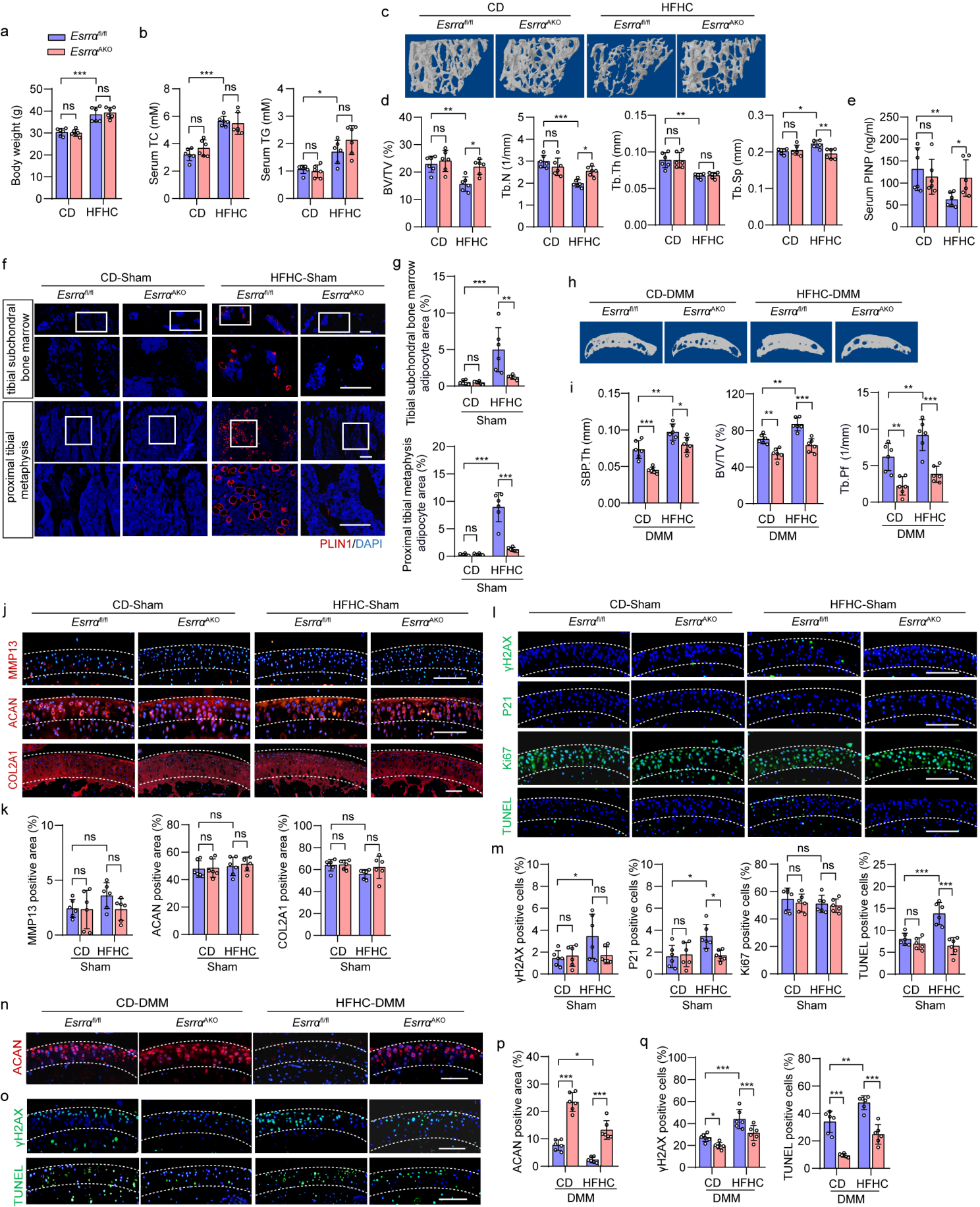


**Supplementary Fig. 2 Loss of adipocyte ESRRA combats bone loss, MAT expansion and osteoarthritis pathogenesis induced by HFHC feeding combined with DMM.**

**a**, Body weights of *Esrra*^fl/fl^ and *Esrra*^AKO^ mice fed a CD or HFHC. **b**, Serum TC and TG levels. **c**, Representative micro-CT images of coronal 3D view of tibial trabecular bone. **d**, Bone histomorphometric of BV/TV, Tb. Th, Tb. N and Tb. Sp. **e**, Serum P1NP levels. **f**, **g**, Representative images (**f**) and quantitative analysis (**g**) of PLIN1^+^ bone marrow adipocytes in the tibial subchondral bone marrow and proximal tibial metaphysis of CD- and HFHC- fed *Esrra*^fl/fl^ and *Esrra*^AKO^ mice following sham surgery. Scale bar, 100 µm. **h**, **i**, Three-dimensional reconstruction of subchondrial bone by microCT (**h**) and quantitative analysis (**i**) of SBP thickness, BV/TV and Tb.Pf of tibial subchondral bone in *Esrra*^fl/fl^ and *Esrra*^AKO^ mice fed a CD or HFHC following DMM surgery. **j**, **k**, Immunostaining (**j**) and quantitative analysis (**k**) of MMP13, ACAN and COL2A1 in mice cartilage. Scale bar, 100 µm. **l**, **m**, Representative fluorescent images of γH2AX, p21, Ki67 and TUNEL (**l**) and quantitative analysis of positive-stained cells (**m**) in the cartilage. Scale bar, 100 µm. **n**-**q**, Immunofluorescence staining (**n**, **o**) and quantification (**p**, **q**) of ACAN, γH2AX and TUNEL within cartilage regions. Scale bar, 100 μm. For all experiments, n = 6 mice per group. Data are shown as mean ± SD. Statistical analysis is performed using two-way ANOVA with post-hoc Turkey’s multiple comparisons test.

**
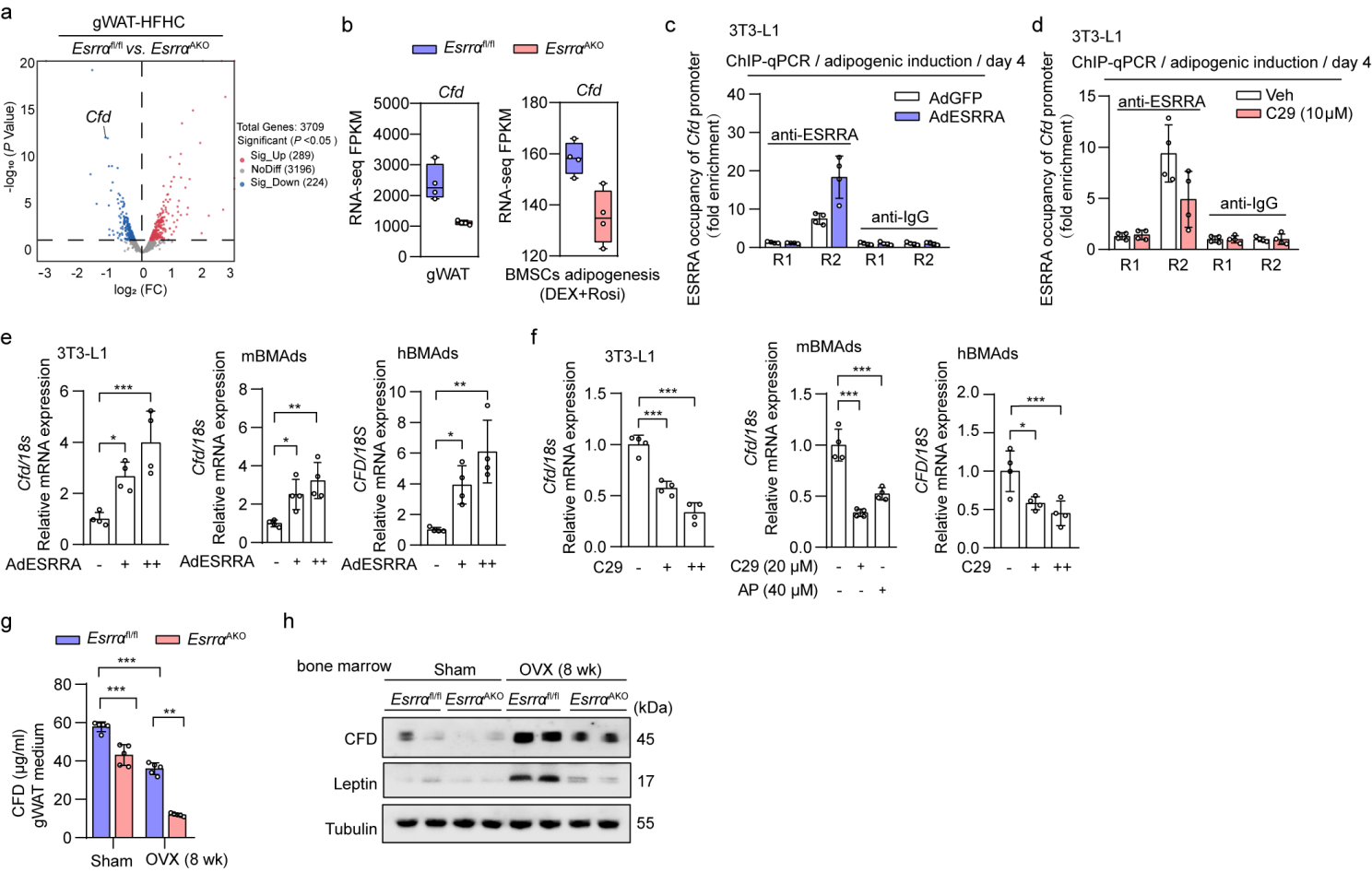
**

**Supplementary Fig. 3 ESRRA directly binds to the *Cfd* promoter and regulates its expression in adipocytes.**

**a**, Volcano plot of transcriptional profiling in gonadal WAT from HFHC-fed *Esrra*^fl/fl^ and *Esrra*^AKO^ mice. Differentially expressed genes identified using DESeq2 (P < 0.05). **b**, Boxplot showing the transcript expression value (FPKM) of *Cfd* based on RNA-seq data. Data are represented as box and whiskers with bars representing maximum and minimum values and with median highlighted as a line (n = 4). **c, d**, ChIP assays with ESRRA-specific antibody or IgG control in 3T3-L1 cells after 4 days of adipogenic induction. Cells were infected with adenoviruses expressing either ESRRA or GFP, or treated with C29 or DMSO (n = 4). **e**, mRNA levels of *Cfd* were measured in mature 3T3-L1 adipocytes, murine and human BMSCs-derived BMAds, which were infected with adenoviruses expressing ESRRA or control GFP for an additional 2 days (n = 4). **f**, mRNA levels of *Cfd* were measured in mature 3T3-L1 adipocytes, mBMAds and hBMAds treated with C29 or AP for 2 days (n = 4). **g**, ELISA analysis of CFD secretion levels from the culture medium of gWAT explants in female *Esrra*^ﬂ/ﬂ^ and *Esrra*^AKO^ mice following sham surgery or ovariectomy (OVX) (n = 5). **h**, Immunoblot analysis of CFD and Leptin in bone marrow from sham-operated and OVX-operated mice. Data are shown as mean ± SD. Statistical analysis is performed using one-way ANOVA followed by Bonferroni’s post hoc tests (**e, f**), two-way ANOVA with post-hoc Turkey’s multiple comparisons test (**g**).


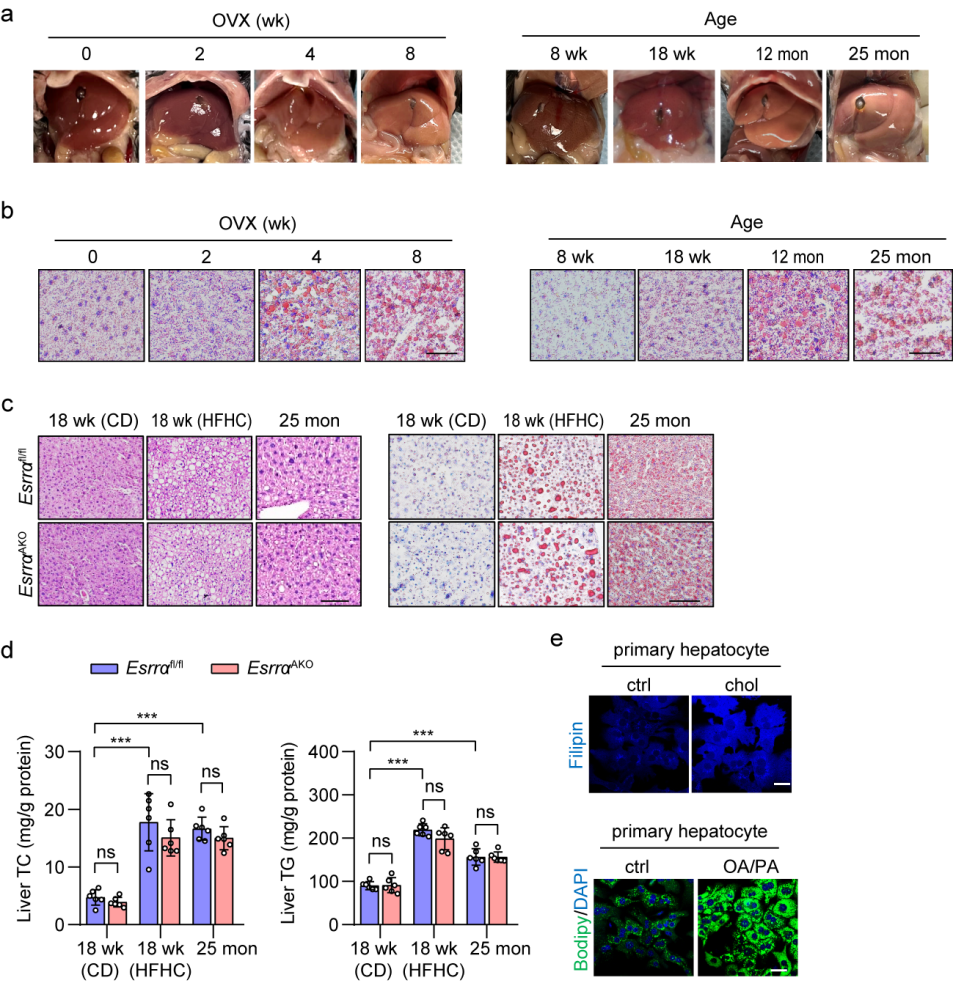


**Supplementary Fig. 4 The severity of hepatic steatosis increases under diverse pathophysiological conditions, and characterization of lipid-overloaded primary hepatocytes.**

**a**, Representative images of liver tissue from female mice at various time points after OVX (left) and male mice across a range of ages (right). **b**, Representative images of Oil Red O-stained liver sections. Scale bar, 50 μm. **c**, Representative images of H&E-stained (left panel) and Oil Red O-stained (right panel) liver sections from *Esrra*^ﬂ/ﬂ^ and *Esrra*^AKO^ mice. Scale bar, 50 μm. **d**, Hepatic TC and TG content from mice as in (**c**). n = 6 mice. **e**, Representative images of primary hepatocytes showing cholesterol (chol) (determined by Filipin staining, blue) and lipid droplets (determined by BODIPY staining, green) via immunofluorescence following treatment with chol or an OA/PA mixture for 24 hours. Scale bar, 10 μm. Data are shown as mean ± SD. Statistical analysis is performed using two-way ANOVA with post-hoc Turkey’s multiple comparisons test.


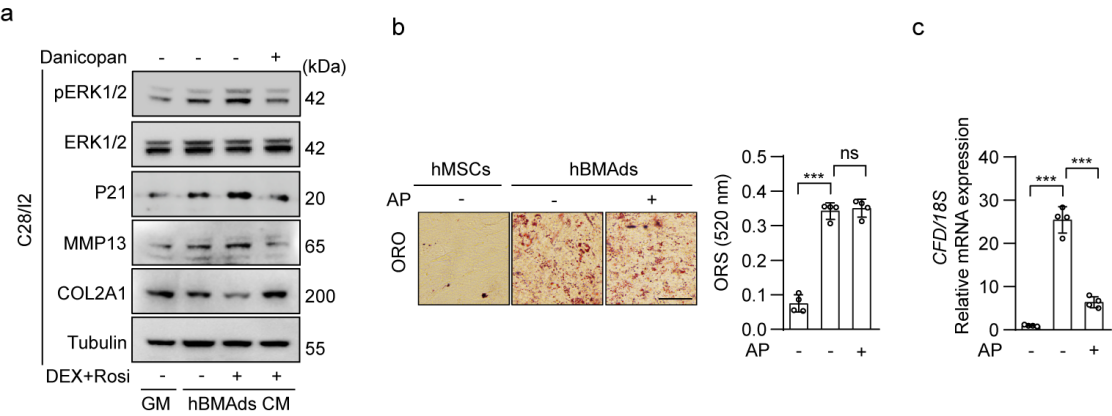


**Supplementary Fig. 5 Danicopan or andrographolide prevents C3-induced human C28/I2 chondrocytes senescence and catabolism by inhibiting CFD.**

**a**, Protein levels of pERK1/2, ERK1/2, P21, MMP13, and COL2A1 in C28/I2 chondrocytes co-cultured with hBMAds CM, in the absence or presence of 10 μM Danicopan. **b**, Representative images (left) and quantification (right) of Oil Red O staining in human BMSCs-derived BMAds treated with 40 μM AP or DMSO for 2 days (n = 4). **c**, *CFD* mRNA levels in hBMAds (n = 4). Data are shown as mean ± SD. Statistical analysis is performed using two-way ANOVA with post-hoc Turkey’s multiple comparisons test.


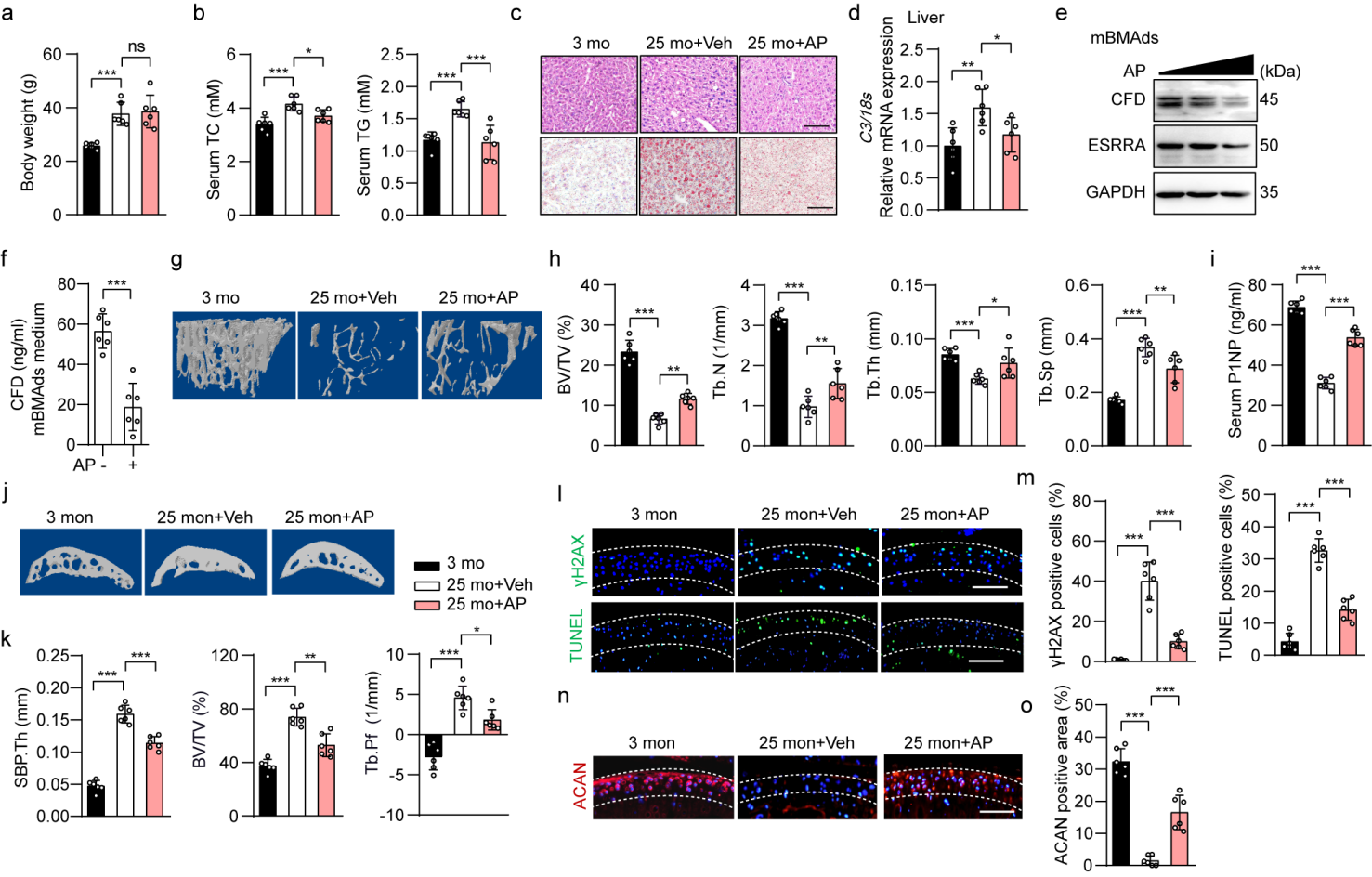


**Supplementary Fig. 6 Oral administration of andrographolide in aged mice attenuates osteoporosis, osteoarthritis and hepatic steatosis.**

**a**, Body weights were measured in 25-month-old male mice following oral administration of AP or vehicle. 3-month-old male mice were used as young controls. **b**, Serum TC and TG levels. **c**, Representative images of H&E-stained (upper panel) and Oil Red O-stained (lower panel) liver sections. Scale bar, 50 μm. **d**, The mRNA expression levels of hepatic C3. **e,** The protein levels of CFD in murine BMAds treated with 20 or 40 μM AP. **f**, ELISA analysis of secreted CFD contents in mBMAds CM (n = 6). **g**, Coronal three-dimensional reconstruction of tibial trabecular bone generated using micro-CT. **h**, Quantitative analysis of BV/TV, Tb. N, Tb. Th and Tb. Sp. **i**, Serum P1NP levels. **j**, Three-dimensional reconstruction of subchondrial bone by microCT. **k,** Quantitative analysis of SBP thickness, BV/TV and Tb.Pf in the medial side of tibial subchondral bone. **l**, **m**, Immunofluorescence staining (**l**) and quantification (**m**) for γH2AX and TUNEL in articular cartilages. Scale bar, 100 μm. **n**,**o**, Immunofluorescence staining (**n**) and quantification (**o**) for ACAN in articular cartilages. Scale bar, 100 μm. For all animal experiments, n = 6 mice per group. Data are shown as mean ± SD. Statistical analysis is performed using unpaired two-tailed Student's *t*-test. (**f**), two-way ANOVA with post-hoc Turkey’s multiple comparisons test (**a, b, d, h, i, k, m, o**).

Supplementary Table S1. Primer sequence of qPCR

| Primer name | Sequence 5’-3’ |
| --- | --- |
| *m18s* | F: TAAGTCCCTGCCCTTTGTACACA |
|  | R: GATCCGAGGGCCTCACTAAAC |
| *mCfd* | F: CATGCTCGGCCCTACATGG |
|  | R: TCCCAATAGGAATACCGGGATT |
| *mMmp3* | F: GTTCTGGGCTATACGAGGGC |
|  | R: TTCTTCACGGTTGCAGGGAG |
| *mMmp9* | F: AGACCTGAAAACCTCCAACCTC |
|  | R: AGACTGCTTCTCTCCCATCA |
| *mMmp13* | F: ATCCAGCTAAGACACAGCAAGCCA |
|  | R: TGGAGCACAAAGGAGTGGTCTCAA |
| *mAdamts4* | F: ATGGCCTCAATCCATCCCAG |
|  | R: GCAAGCAGGGTTGGAATCTTTG |
| *mAdamts5* | F: GCCATTGTAATAACCCTGCACC |
|  | R: TCAGTCCCATCCGTAACCTTTG |
| *mIl-6* | F: ATCCAGTTGCCTTCTTGGGACTGA |
|  | R: TAAGCCTCCGACTTGTGAAGTGGT |
| *mP16* | F: GTCGCAGGTTCTTGGTCACT |
|  | R: CATGTTCACGAAAGCCAGAGC |
| *mP21* | F: CCTGGTGATGTCCGACCTG |
|  | R: CCATGAGCGCATCGCAATC |
| *mP53* | F: GGCGTAAACGCTTCGAGATG |
|  | R: CTTCAGGTAGCTGGAGTGAGC |
| *mC3* | F: CCAGCTCCCCATTAGCTCTG |
|  | R: GCACTTGCCTCTTTAGGAAGTC |
| *hCfd* | F: CTCCAAGCGCCTGTACGACGT |
|  | R: CCAGTGTGGCCTTCTCCGACA |

Supplementary Table S2. Primer sequence of promoter vector

| Primer name | Sequence 5’-3’ |
| --- | --- |
| m*Cfd* promoter-WT | F: CGGGGTACCGATGGGCGAGTCAGATGATTCTA  R: CCCAAGCTTCAGGCTCTCAGGGCTGTTTT |
| m*Cfd* promoter-ΔR2 | F: CGGGGTACCCTGCGCTAAAGGGCTTCTGA  R: CCCAAGCTTCAGGCTCTCAGGGCTGTTTT |
| m*Cfd* promoter-MutantS2 | F: CggggtaccAGCCATGaTgAtGAAGGAGAC  R: cccaagcttGTACACGGAGCTGTGCATTCT |

Supplementary Table S3. Primer sequence of ChIP

| Primer name | Sequence 5’-3’ |
| --- | --- |
| m*Cfd* promoter- region1  m*Cfd* promoter- region2 | F: TAGAACCCACCCTCAGGATTA  R: CTCACCAGGCTCTTCCCTATT  F: AATAAGCTAGGACTGGTGAAGTGG  R: TTTATGTACTGTGTGGGTAAGTGT |
